# The impact of DICOM import/export on radiotherapy structures in commercial systems

**DOI:** 10.1016/j.phro.2026.100981

**Published:** 2026-04-26

**Authors:** Mark J. Gooding, Annamieke Koops, Ciaran Malone, David Nash, Maxwell Robinson, Daniel Rossiter, Rita Simões, Christina Skourou, Georgios Tsekas, Kieran Venner, Djamal Boukerroui

**Affiliations:** a Inpictura Limited, Abingdon, United Kingdom; b Division of Cancer Sciences, Faculty of Biology, Medicine and Health, The University of Manchester, Manchester, United Kingdom; c Department of Radiation Oncology, University Medical Center Groningen, Groningen, Netherlands; d St. Luke’s Radiation Oncology Network, St. Luke’s Hospital, Dublin, Ireland; e Erasmus MC Cancer Institute, Department of Radiotherapy, University Medical Center Rotterdam, Rotterdam, Netherlands; f Department of Medical Physics, Portsmouth Hospitals University NHS Trust, Portsmouth, United Kingdom; g Department of Oncology, Oxford University Hospitals NHS Trust, Oxford, United Kingdom; h Department of Radiation Oncology, The Netherlands Cancer Institute, Amsterdam, the Netherlands

**Keywords:** Contouring, Contour similarity measures, DICOM, Auto-contouring, Contour representation, Commercial software

## Abstract

Background and Purpose: To investigate the impact that loading contours into an internal representation and saving them back to DICOM format has on contour position within commercially available contouring systems.

Materials and Methods: Two DICOM RT Structure Sets (RTSS) were loaded into 12 different commercial contour editing systems. Small non-editing actions were taken to prompt the systems to convert the structures to their internal representation. Contours were exported again as DICOM RTSS. The exported contours were compared to the original contours. The impact on contour similarity measures was quantified by their calculation against a common reference set for both the original and the re-exported contours.

Results: Average distances up to 0.23 mm were observed between the original and re-exported contours on thoracic data for some systems, with maximum distances up to 37.7 mm resulting from topological changes. Contour boundary changes impacted contouring similarity measures up to 2.6%, 29%, 35% and 21% for volumetric Dice Similarity Coefficient, 2D Mean Surface Distance, 3D Mean Surface Distance, and Hausdorff Distance, respectively. Notably, Surface Dice Similarity Coefficient and Normalised Added Path Length varied up to 96% and 97% respectively at low tolerances. Other systems preserved the original contours.

Conclusions: Caution should be employed when comparing contouring accuracy measures between studies or within multi-centre evaluations if different contouring systems have been used. For monitoring auto-contouring in clinical practice, differences between automatic and clinical contours smaller than one quarter of the in-plane voxel size can be disregarded, as possibly reflecting software-related effects rather than intentional editing.

## Highlights

• Loading and exporting DICOM RTSS in commercial systems may move contour boundaries.
• Topological differences can be introduced by automatic disconnected regions removal.
• Caution should be applied if comparing contours after export from different systems.
• Export difference between systems may hinder measurement comparison between studies.
• A tolerance of at least one-quarter pixel should be used when monitoring AI contours.

## Graphical abstract

**Figure:**
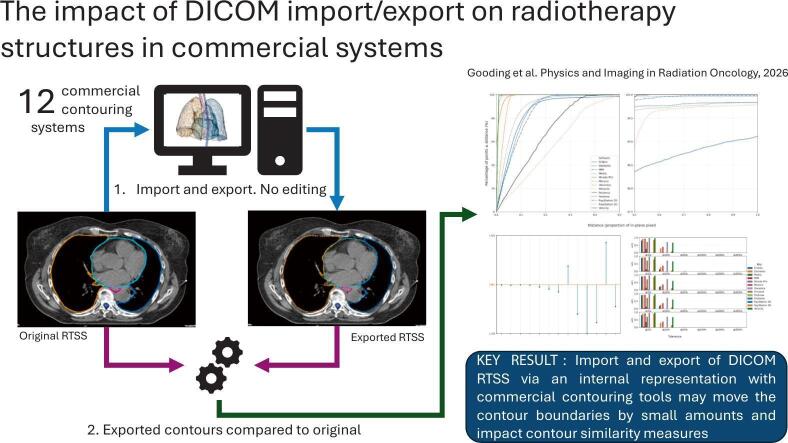

## 1 Introduction

Over the past decade, automatic normal anatomy contouring using deep learning methods has become ubiquitous in radiotherapy. This development has been backed by research comparing automatically generated contours with independently outlined clinical reference contours using contour similarity measures (CSMs) (e.g. [1], [2], [3]). That same type of assessment is also commonly performed when comparing or commissioning automatic contouring systems for clinical use (e.g. [4], [5], [6]). In that context, studies may also evaluate the amount of editing required to make the automatic contours clinically acceptable, and the same types of CSM are computed between the automatic contours and the edited ones (e.g. [7], [8], [9]). This approach of measuring the difference between the automatic contours and their edited versions can also be used for monitoring the use of automatic contouring software in clinical practice (e.g. [10], [11], [12]), where changes in the contour boundary can be considered as clinical corrections.

An inherent assumption is that the only sources of difference between the contours are intended algorithmic differences (such as different vendors) or the editing being performed on the automatic contours. However, it has been shown for other measurements that differences may arise as a consequence of the software used in processing. For example, studies have found substantial differences, of up to 22%, in volume calculation between different Treatment Planning Systems (TPS), particularly as a consequence of how the systems treat the end slices of a structure [13], [14], [15], [16], [17]. This can affect Dose-Volume Histogram (DVH) calculation accuracy. It was observed, for one TPS [18], that the volume of the end slices was fully accounted for, but not the dose within this region. Such differences in DVH calculation between systems [15], [18], stem from both volume estimation and, more critically, how dose is sampled. This can have clinical implications, particularly for the target region, if the clinical criteria appear met in one system and not in another [19]. The interpretation of structure boundaries has also been found to affect margin application, with differences between systems [14], [17], [20], [21]. This, in part, is a consequence of how structures are represented. The DICOM Radiotherapy Structure Set (RTSS) defines structures as stacks of 2D polygons, but many software applications convert these into 3D representations internally. This representational choice has been found to be a source of difference in CSM calculations in different systems [22].

Differences in CSMs computation only matter when comparing measurements generated by different systems. The results should remain consistent if a single system is used to calculate these measures within a study. However, in cases where contours are transferred between systems, it is unclear how the choice of contour-editing system itself might influence the outcomes beyond the intended changes. This study aims to address the question; Does the contour editing system impact the contour boundary position independently of any editing performed? Are errors, or bias, in quantification and measurement of editing introduced through loading and saving of contours?

## 2 Materials and methods

Two publicly available contour sets [23], [24], previously used to study the implementation accuracy of CSMs [22], were used in this investigation. Both datasets consist of pairs of contours labelled as ‘reference’ and ‘test’ in DICOM RTSS format. The first dataset consists of six pairs of synthetic analytically defined geometric structures with variation in vertex spacing [25], including; maximally sparse cuboids represented only by vertices in the corners of each in-plane square (Objects A and B), densely sampled cuboids (Objects C and D), and octahedrons (sparsely sampled Object E, densely sampled Object F). This dataset was generated using open-source code [26]. The second dataset is an example case from the thoracic contouring challenge [27], incorporating Heart, Lungs (Left & Right), Esophagus and SpinalCord structures. The ‘test’ contours were generated with a commercial automatic contouring system (DLCExpert, Mirada Medical, Oxford, UK); The ‘reference’ set consisted of manually drawn contours, exported from a commercial contouring workstation (Mim Maestro, MIM Software/GE Healthcare, Cleveland, USA).

Twelve commercial contouring packages were used in this investigation. Each test contour was loaded into each contour editing package. An editing action was taken to prompt the software to convert the RTSS into its native internal representation while not intentionally modifying the contour’s boundary. The unedited structures were then exported again as RTSS. Table 1 outlines the manufacturer information and the non-editing action(s), that were performed with each system. This process was carried out using clinical software within various institutions using the system configuration used clinically.

**Table 1:** Contouring software assessed and action taken to encourage the software to convert to its internal representation prior to export.

Name used in results	Software version and manufacturer	Actions performed
Eclipse	Eclipse v18.0.567 Varian Medical Systems/Siemens, Palo Alto, USA	Painted inside each structure
Elements	Elements v4.5 Brainlab Medical Solutions, Munich, Germany	Imported to Elements via Eclipse (unaltered). Painted inside each structure within Elements
Mediq	Mediq v1.4.0 Synaptiq, Cluj, Romania	Painted inside each structure
MIM	MIM Maestro v7.4.200 MIM Software/GE Healthcare, Cleveland, USA	Painted inside each structure
MiradaRTx	Mirada RTx v1.8.8 Mirada Medical, Oxford, UK	Painted inside each structure
Monaco	Monaco v6.2.2 Elekta Solutions, Stockholm, Sweden	Painted inside each structure
Oncentra	Oncentra v4.5.3.15 Nucletron/Elekta Solutions, Stockholm, Sweden	Painted inside each structure
Pinnacle	Pinnacle v16 Koninklijke Philips NV, Eindhoven, Netherlands	Painted inside each structure on each slice
ProKnow	ProKnow DS v2.0.7.2 Elekta Solutions, Stockholm, Sweden	Painted inside each structure
ProSoma	ProSoma v42B410 MedCom, Darmstadt, Germany	Copied contours
Raystation2D	RayStation v2025a [17.0.1.113] Raysearch Laboratories, Stockholm, Sweden	Painted inside each structure using 2D brush
Raystation3D		Painted inside each structure using 3D brush
Velocity	Velocity v4.1.4 Varian Medical Systems/Siemens, Palo Alto, USA	Painted inside each structure

Changes in contours were assessed by quantifying the distance from the original RTSS polygon vertices to the nearest location, slice-by,-slice on the re-exported contour. The maximum, mean and histogram of distances were summarized for each structure. Additionally, the number of vertices in the original and re-exported contours were recorded. These calculations were computed using the RTSS directly. Duplicate vertices and degenerate contours (with zero volume) were removed from both original and re-exported data prior to this analysis, so as not to penalise systems that performed this operation during re-export.

A range of CSMs were calculated between the re-exported test contours and the corresponding reference RTSS to determine any impact on these measures. The reference RTSS were used directly in their original form without loading into the contouring software. The same measures were also calculated using the original test contours for relative comparison. The following measures were used: Normalised APL (nAPL) [25], [28], Volumetric Dice Similarity Coefficient (vDSC), Hausdorff Distance in 2D (2DHD), 95% HD in 2D (95%2DHD), Mean average (surface) distance in 2D (2DMSD), Median average distance in 2D (50%2DHD), Surface Dice Similarity Coefficient (sDSC) [3], and Mean surface distance in 3D (3DMSD).

Calculation of nAPL, vDSC, 2DHD, 95%2DHD, 2DMSD, and 50%2DHD was performed directly on the RTSS using the definitions and open-source implementation described in [29]. This implementation was validated using the analytic data described in [25]. The sDSC and 3DMSD measures were computed using the open-source implementation described in [3] which uses a mesh-based representation. Therefore, the contours were converted into an implicit representation at CT image resolution using a distance transform and a mesh was extracted with sub-voxel accuracy using the Marching Cubes algorithm.

For nAPL and sDSC, calculations were performed with tolerances of 1%, 10%, 50%, 100%, 200% and 300% of the in-plane voxel size. A tolerance relative to the voxel size was used, both to provide an image resolution-based interpretation to the tolerance used, and because the datasets did not share the same resolution.

The reproducibility of the results was investigated, to a limited extent, with two institutions performing the experiment with the same software version of MiradaRTx. Furthermore, the experiment was repeated for Eclipse, within a single centre, using several non-editing actions; painting within the structure, duplicating the structure set, and duplicating each structure within a duplicated structure set.

## 3 Results

No difference in results was observed between different institutions repeating the test with the same software (MiradaRTx), or different non-editing actions being performed in Eclipse.

**Fig. 1:**
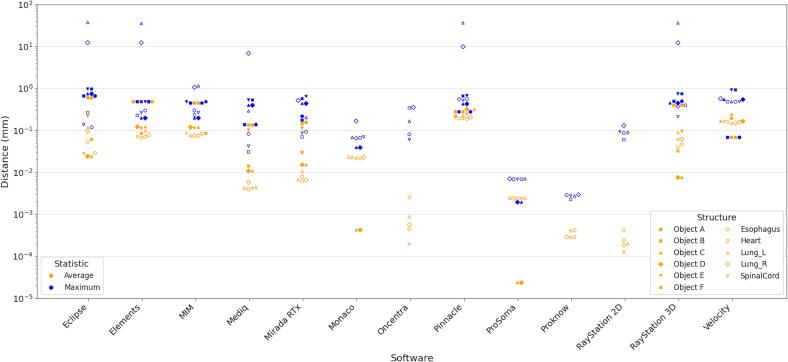
Average and Maximum distance error by Manufacturer and Structure. Where markers are not shown (Object A-F for Oncentra, Proknow, and RayStation 2D, and Object A, B, E, and F for Monaco and Prosoma), the values are below 10^−5^ mm indicating the DICOM polygon point positions have been preserved.

Comparison of the re-exported RTSS with the original RTSS found average distances in the range 0.00–0.61 mm for the synthetic dataset over all structures, and 0.00–0.23 mm for the thoracic challenge dataset. Maximum distances were in the range 0.00–0.98 mm and 0.00–37.67 mm respectively for these datasets. The average and maximum distances are shown for each structure and manufacturer in Fig. 1. Markers are not shown on Fig. 1 where the distance of the contour from the original contour was less that 10^−5^ mm. This was the case for both the maximum and average distances for Oncentra, Proknow, and RayStation2D for all structures from the synthetic dataset and for Objects A, B, E and F for Monaco and Prosoma. Full quantitative results corresponding to Fig. 1, and including the number of contour vertices, are given in the Supplementary Material S1.

The number of contour vertices varied from the original in the re-exported contours, with only ProSoma and Monaco producing contours with an identical number of points. Oncentra, ProKnow, and RayStation2D had approximately the same number of points for most structures. ProKnow sub-sampled the more densely sampled synthetic data, while preserving the position. RayStation3D and Velocity had generally sparser sampling. Eclipse, Elements, Mediq, MIM, and MiradaRTx produced more densely sampled contours, except for the Esophagus and SpinalCord which had substantially fewer points than the original contours in all but the latter system.

**Fig. 2:**
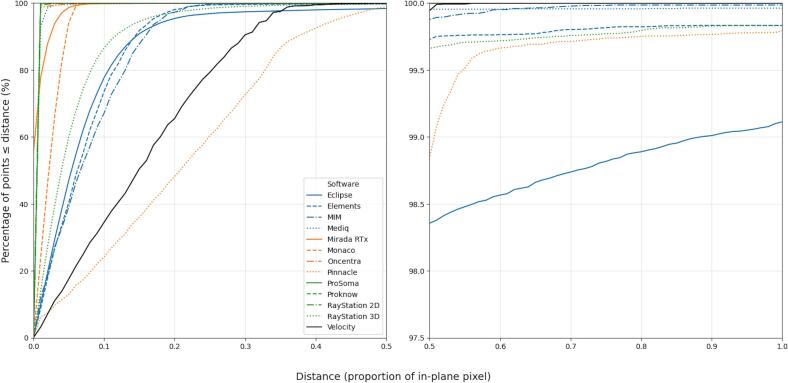
Cumulative Distance Distribution for the Right Lung. The plot shows the percentage of RTSS vertices from the original contour that have been displaced by more than the distance indicated on the x-axis. The x‑axis distances are given relative to the in‑plane CT image voxel size of the original dataset. The plot is split to show the tail of the distribution for distances greater than half an in-plane CT voxel size, noting the change of the y-axis scale.

Cumulative histograms of the distances from the original contour vertices to the closest location on the re-exported contour are shown for the Right Lung in Fig. 2. It shows the percentage of the original contour vertices that are less than a particular distance away from the new contour. The distances on the x-axis are shown as a proportion of the in-plane image voxel size. The x-axis is split to show the tail of the distributions at a different y-axis scale. Editing systems can be broadly grouped into four categories; those for which the distances from the original contour vertices to the re-exported contour are negligible, with 100% of vertices less than 0.01 voxels from the new contour (Oncentra, ProKnow, ProSoma, Raystation2D), those for which there is some minor vertex movement, with 100% of vertices less than 0.1 voxels away (Mediq, MiradaRTx, Monaco), those that converge rapidly to their asymptote with most vertices less than 0.2 voxels from the new contour (Eclipse, Elements, MIM, RayStation3D), and those which converge slowly to their asymptote (Pinnacle, Velocity). Notably, some systems (Eclipse, Elements, Mediq, Pinnacle, RayStation3D) have a non-negligible proportion of vertices from which the distance to the new contour is greater than a voxel. Plots for all structures are given in the Supplementary Material S2. Additionally, quantitative results showing the distances starting from the re-exported contour vertices back to the original polygon are included in the Supplementary Material S1.

**Fig. 3:**
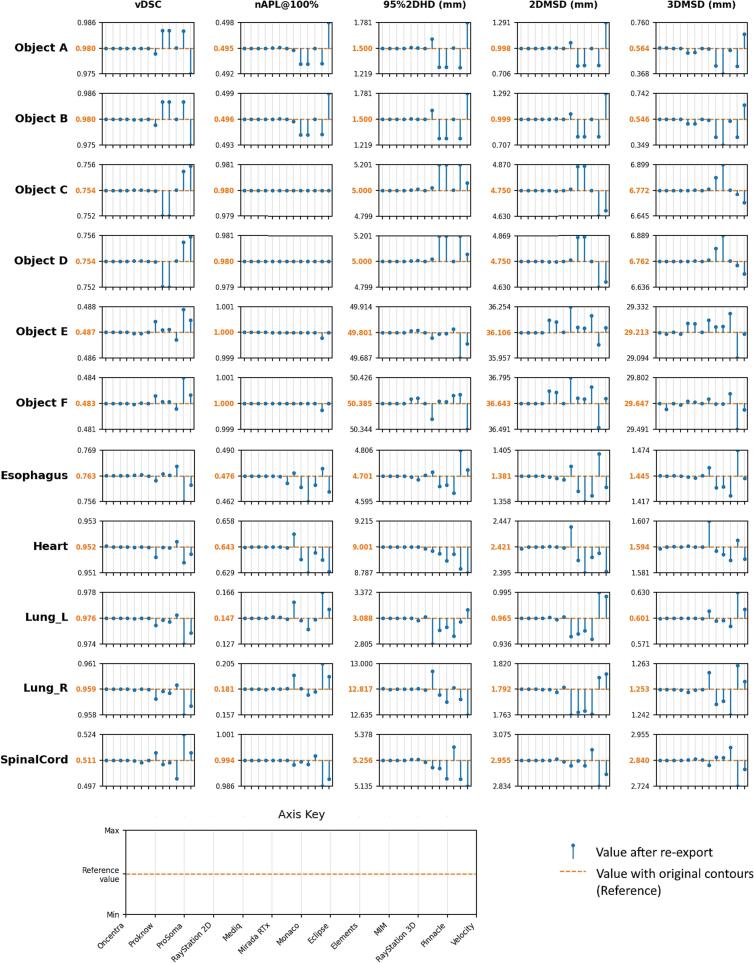
Quantitative similarity measure values for the re-exported contours relative to the measure calculated using the original data. Each plot is scaled such that the reference value, calculated in using the original contours, is shown centrally on the y-axis. The range of the y-axis is scaled symmetrically to include the minimum and/or maximum value. Systems have been sorted according to a rough classification into groups according to amount of contour position displacement using the cumulative histogram (as in Fig. 2). Those displacing the contour least are shown first in this figure.

The values for selected CSMs for the re-exported contours are shown in Fig. 3 relative to the same measure when calculated for the original test contours. This representation is intended to give insight as to the relative impact on the measure after re-exporting from the different systems rather than absolute performance. Not all measures are shown for the sake of figure clarity and similar performance was observed between related measures. Tables of results for all measures are given in the Supplementary Material S3.

Notable differences were seen between systems for Objects A and B with the maximum discrepancy being > 15% for all distance measures, and up to 35% for the 50%2DHD. The results deviated by less than 2.6% of the reference values for vDSC, with the greatest differences being seen in the SpinalCord (Reference: 0.511, Minimum: 0.501 Raysearch3D, Maximum: 0.524 Pinnacle). Similarly, the greatest deviation of the sDSC@100% measure was found in the SpinalCord (Reference: 0.011, Minimum: 0.010 for Raysearch3D, Maximum: 0.018 for Pinnacle). The systems showed greatest variation for nAPL@100% in the lungs (Lung_L; Reference: 0.147, Minimum: 0.138 for MIM, Maximum: 0.159 for Pinnacle. Lung_R; Reference 0.181, Minimum: 0.175 for MIM, Maximum: 2.05 for Pinnacle).

**Fig. 4:**
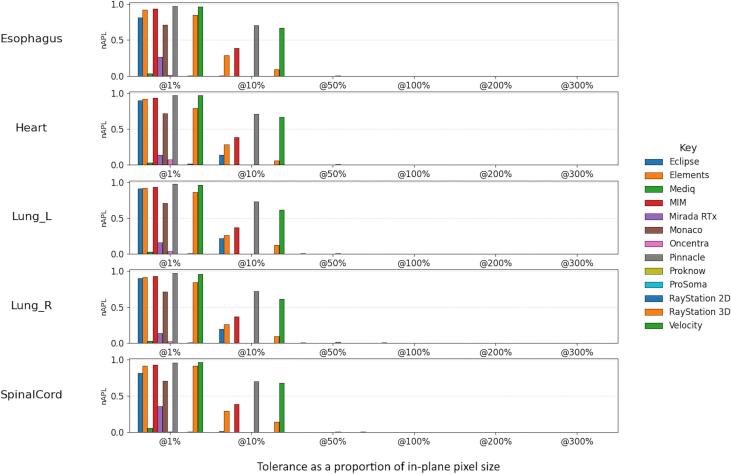
nAPL at various tolerances for the thoracic dataset between the re-exported contours and the original contour. The tolerance is expressed relative to the in-plane voxel spacing in percentage. A nAPL values of 1 indicates that re-exported contour is outside the tolerance everywhere, while a value of 0 indicates it is within the tolerance everywhere.

**Fig. 5:**
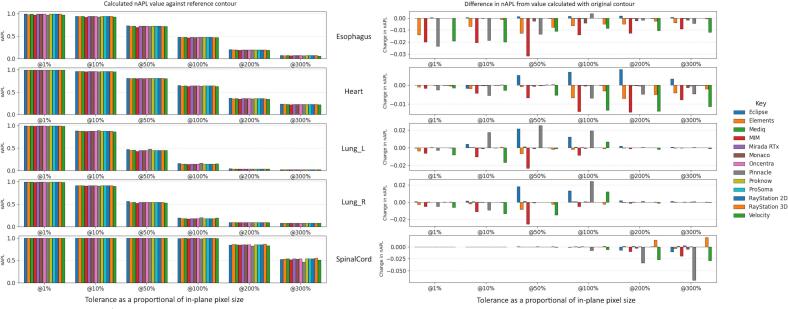
Value of nAPL (left) and Difference in nAPL value from that calculated with the original contours (left) for the structures of the thoracic case when calculated with respect to the reference contour.

Fig. 4 shows the nAPL values for the structures of the thoracic contouring case for the re-exported contours when calculated against the original test contours at different tolerance. Here, the expected value is 0 for all tolerances, as the contours should be identical to the original test contours. Noting that nAPL is bounded from 0 to 1, differences from the expected value of greater than 0.1 can be observed for 8 and 4–6 of the systems (depending on structure) for the 1% and 10% tolerance respectively. The left-hand subplot of Fig. 5 shows the nAPL values between the re-exported test contours and the reference structure for the thoracic structures. The difference from that calculated between the original test contours and the reference are shown on the right-hand subplot. The magnitudes of the difference were smaller in this scenario, with a maximum absolute difference of 0.069 being seen for the SpinalCord at 300% tolerance for the re-exported contours from Pinnacle. Equivalent plots for Figs. 4 and 5 are shown for sDSC in the Supplemental Material S4.

## 4 Discussion

This study sought to investigate the impact that loading and re-saving contours in commercial contour editing systems can have on the contour boundary position, and consequently contouring similarity measures calculated from them. The key finding is that the process of re-exporting can displace contours and even change their topology.

For many systems, the average distance between the original and the re-exported contours was non-negligible, although less than the in-plane voxel size. Such differences can be explained by conversion of the slice-based polygon DICOM contours to a volumetric representation (e.g. mask or mesh based), for which the reverse transformation does not preserve the initial boundary. This can be seen most clearly for the sparsely sampled synthetic shapes (Objects A and B), where the average distance is 0.6 mm for one system. However, some systems appear to preserve the original contour locations, resulting in tiny, or no, difference (Oncentra, ProKnow, ProSoma, RayStation 2D, Monaco). These small differences can be explained by the different number of decimal places used in the “DecimalString” representation of the vertex locations by each system (Supplemental Material S5).

The large (directional) 2DHD, of up to 37.7 mm for the Lung structures from the challenge data, calculated between the original contours to the re-exported contours, without similarly large 2DHD in the opposite direction, indicates a change in topology. Visual inspection of the data confirmed the finding consisting of small disconnected regions being removed automatically on re-exporting by some systems. These topological changes are also reflected in the cumulative distribution (Fig. 2), where the percentage of vertices is less than 100 at a distance of one in-plane voxel. Note that topological changes resulting from degenerate contours were not included in this analysis, and some variation was observed in how systems handled such cases.

Apart from topological changes, the contour boundaries were typically displaced by less than one in-plane voxel. This is unlikely to have a significant clinical impact in planning but may impact the apparent dosimetry near high dose gradients if different systems are used to assess the dose volume. Such changes in contouring position may also in part explain DVH differences previously observed [15], [18], [19].

CSMs are mainly used in the assessment of contouring accuracy for the development or selection of auto-contouring systems. Although the absolute values of CSMs may be impacted, any comparison of contouring systems should still be valid if a single contouring system is used (for each of the test or reference, and for all structures). However, in a multi-centre study, or when comparing between studies, results may not be directly comparable if different contouring systems have been used (Fig. 3). The difference in vDSC observed, of up to 0.01 (1% of the range of the measure) may be sufficient to draw incorrect conclusions of relative performance for systems that perform similarly.

More recently, CSMs have been used for monitoring the use of automatic contouring systems [12], [30], [31], [32]. Here, the impact could be more significant, particularly for structures where automatic contours are considered largely accurate and small edits are being considered. The clinical contour should be identical to the initial automatic contour if it has not been edited, with nAPL of 0, sDSC of 1, and MSD of 0 mm. Since any difference between the contours should be interpreted as a manual edit, a very small tolerance should be used for sDSC and nAPL in this context. However, the results of this study indicate that re-exporting from a manual contouring system may move the contour boundary, and, in practice, an MSD of up to 0.25 mm might be reported, depending on the system, even if the clinical contour was re-exported even without any editing being performed. For nAPL and sDSC, this difference could make it appear all of the contour has been edited when a very small tolerance is used (Fig. 4). Therefore, a tolerance of quarter to half of an in-plane voxel size seems more appropriate to detect actual clinical edits to a contour in a manner that is robust to different editing software, balancing the risk of missing small intentional edits with the risk of including software induced changes when monitoring the use of auto-contouring.

This study has several limitations, the most significant of which is that some action was taken within each manual contouring software. These actions were intended to prompt the software to convert the loaded structure into its native contour representation without actually modifying the contours. This does not reflect an actual clinical workflow. Although unlikely, the operator may have accidentally changed the contour, or the operation may not have triggered the software to use its internal representation. Additionally, the impact of the image resolution on the error was not fully investigated (a preliminary post-hoc investigation is given in Supplemental Material S6), weakening the conclusions that can be drawn about representations used in each software and consequently the appropriate tolerance for APL and sDSC to determine editing. The impact of repeatedly loading and re-saving was not investigated, and thus it is unknown if contours will move further, or whether each system reaches a stable state where the contour saved from the system will be preserved if re-exported again. This scenario is likely to happen in clinical radiotherapy workflows involving different personnel checking different structures or performing different aspect of the treatment planning. Lastly, the limited dataset only demonstrates that contours may be moved and does not indicate of how much this may vary across datasets.

While the effect of re-exporting contours was found to be small within this study, save topological changes, this study highlights the need to be aware of the impact data transfer can have on contouring measures. Caution is recommended when comparing differences in contouring accuracy where different viewing/editing software has been used. Particular caution should be taken for non-linear measures such as the nAPL and sDSC where small differences on the tolerance used could have a major difference on the reported values. For monitoring of auto-contouring in clinical practice, changes in the contour smaller than one quarter of the in-plane voxel size should not be considered as clinical edits.

## CRediT authorship contribution statement

**Mark J. Gooding:** Conceptualization, Formal analysis, Investigation, Methodology, Project administration, Software, Validation, Visualization, Writing – original draft. **Annamieke Koops:** Investigation, Writing – review & editing. **Ciaran Malone:** Investigation, Writing – review & editing. **David Nash:** Investigation. **Maxwell Robinson:** Investigation. **Daniel Rossiter:** Investigation. **Rita Simões:** Investigation. **Christina Skourou:** Investigation, Writing – review & editing. **Georgios Tsekas:** Investigation. **Kieran Venner:** Investigation. **Djamal Boukerroui:** Investigation, Methodology, Writing – review & editing.

## Declaration of competing interest

The authors declare the following financial interests/personal relationships which may be considered as potential competing interests: M. Gooding and D. Boukerroui are shareholders and employees of Inpictura Ltd. Inpictura Ltd produces software for analysis of automatic contouring. All other authors have no competing interests to declare.
