# Supplementary material for: The impact of DICOM import/export on radiotherapy structures in commercial systems
Source: Phys Imaging Radiat Oncol. 2026 Apr 26;39:100981. doi: 10.1016/j.phro.2026.100981 (PMC13157206; doi:10.1016/j.phro.2026.100981)
Supplement: Supplementary Data 1 [file mmc1.pdf]

The impact of loading and saving on contour position and contouring measures – Supplementary Material

Mark J. Gooding, Annamieke Koops, Ciaran Malone, David Nash, Maxwell Robinson, Daniel Rossiter, Rita Simões, Christina Skourou, Georgios Tsekas, Kieran Venner, Djamal Boukerroui

S1: Difference from original contour

These tables give the measurements of the Mean and Maximum 2D distance of the original contour vertices to the re-exported contour. The distance from the re-exported contour vertices back to the original contour is also provided, as large discrepancies indicate where there have been topological changes (i.e. deleting of small area).

Eclipse	Distance from original to re-exported (mm)		Distance from re-exported to original (mm)		Number of RTSS vertices	
Structure	Average	Maximum	Average	Maximum	Original	Re-exported
Object A	0.61	0.66	0.07	0.20	100	5200
Object B	0.61	0.66	0.07	0.20	100	5200
Object C	0.02	0.75	0.01	0.06	14420	7140
Object D	0.02	0.75	0.01	0.06	14420	7140
Object E	0.22†	0.98†	0.04	0.09	2400	20080
Object F	0.06†	0.98†	0.04	0.09	20000	20080
Esophagus	0.03	0.12	0.03	0.13	13217	4736
Heart	0.05	0.27	0.06	0.26	14415	6314
Lung_L	0.12	37.67	0.06	0.69	35080	16602
Lung_R	0.10	12.36	0.06	0.91	39065	19468
SpinalCord	0.03	0.14	0.03	0.15	10744	3852

† Contours from the 2 end slices were deleted, thus the calculation does not include distances for these slices. The two remove slices represented very small islands at the extreme of an octahedron.

Elements	Distance from original to re-exported (mm)		Distance from re-exported to original (mm)		Number of RTSS vertices	

Structure	Average	Maximum	Average	Maximum	Original	Re-exported
Object A	0.48	0.48	0.25	0.33	100	300
Object B	0.48	0.48	0.25	0.33	100	300
Object C	0.12	0.20	0.10	0.20	14420	420
Object D	0.12	0.20	0.10	0.20	14420	420
Object E	0.11	0.48	0.09	0.34	2400	81504
Object F	0.08	0.48	0.09	0.34	20000	81504
Esophagus	0.07	0.30	0.08	0.25	13217	6785
Heart	0.07	0.23	0.08	0.24	14415	17977
Lung_L	0.10	36.27	0.08	0.35	35080	49825
Lung_R	0.08	12.34	0.08	0.37	39065	58377
SpinalCord	0.07	0.26	0.08	0.25	10744	5594

Mediq	Distance from original to re-exported (mm)		Distance from re-exported to original (mm)		Number of RTSS vertices	
Structure	Average	Maximum	Average	Maximum	Original	Re-exported
Object A	0.14	0.14	4×10^{-3}	6×10^{-3}	100	10500
Object B	0.14	0.14	4×10^{-3}	6×10^{-3}	100	10500
Object C	0.01	0.40	4×10^{-3}	6×10^{-3}	14420	14280
Object D	0.01	0.40	4×10^{-3}	6×10^{-3}	14420	14280
Object E	0.10	0.53	3×10^{-3}	5×10^{-3}	2400	36314
Object F	0.01	0.53	3×10^{-3}	5×10^{-3}	20000	36314
Esophagus	4×10^{-3}	0.08	3×10^{-3}	7×10^{-3}	13217	4432
Heart	4×10^{-3}	0.03	3×10^{-3}	8×10^{-3}	14415	12007
Lung_L	4×10^{-3}	0.29	3×10^{-3}	7×10^{-3}	35080	32046
Lung_R	6×10^{-3}	6.91	3×10^{-3}	8×10^{-3}	39065	37367
SpinalCord	4×10^{-3}	0.04	3×10^{-3}	7×10^{-3}	10744	3591

MIM	Distance from original to re-exported (mm)		Distance from re-exported to original (mm)		Number of RTSS vertices	
Structure	Average	Maximum	Average	Maximum	Original	Re-exported
Object A	0.45	0.45	0.20	0.21	100	200
Object B	0.45	0.45	0.20	0.21	100	200
Object C	0.12	0.20	0.12	0.20	14420	280
Object D	0.12	0.20	0.12	0.20	14420	280
Object E	0.11	0.48	0.08	0.17	2400	81504
Object F	0.09	0.48	0.08	0.17	20000	81504
Esophagus	0.08	0.30	0.10	0.24	13217	6887
Heart	0.08	0.24	0.10	0.24	14415	18636
Lung_L	0.08	1.17	0.10	0.55	35080	52183
Lung_R	0.08	1.07	0.10	0.52	39065	61967
SpinalCord	0.08	0.26	0.10	0.24	10744	5643

Mirada	Distance from original to re-exported (mm)		Distance from re-exported to original (mm)		Number of RTSS vertices	

Structure	Average	Maximum	Average	Maximum	Original	Re-exported
Object A	0.15	0.22	0.02	0.13	100	609
Object B	0.15	0.18	0.01	0.07	100	573
Object C	0.02	0.45	0.08	0.25	14420	1187
Object D	0.02	0.45	0.08	0.25	14420	1179
Object E	0.11	0.65	0.03	0.27	2400	43888
Object F	0.03	0.58	0.03	0.27	20000	44574
Esophagus	0.01	0.09	0.01	0.09	13217	13140
Heart	0.01	0.07	0.01	0.07	14415	14407
Lung_L	0.01	0.20	0.01	0.16	35080	35067
Lung_R	0.01	0.52	0.01	0.55	39065	39055
SpinalCord	0.01	0.09	0.01	0.08	10744	10769

Monaco	Distance from original to re-exported (mm)		Distance from re-exported to original (mm)		Number of RTSS vertices	
Structure	Average	Maximum	Average	Maximum	Original	Re-exported
Object A	0	0	0	0	100	100
Object B	0	0	0	0	100	100
Object C	$4x10^{-4}$	0.04	0	0	14420	14420
Object D	$4x10^{-4}$	0.04	0	0	14420	14420
Object E	$9x10^{-16}$	0	$9x10^{-16}$	0	2400	2400
Object F	0	0	0	0	20000	20000
Esophagus	0.02	0.07	0.02	0.07	13217	13217
Heart	0.02	0.07	0.02	0.07	14415	14415
Lung_L	0.02	0.07†	0.02	0.07†	35080	35080
Lung_R	0.02	0.17	0.02	0.07	39065	39065
SpinalCord	0.02	0.07	0.02	0.07	10744	10744

†Degenerate contour had been generated by Monaco. Points were included when going from the original to the re-exported by not vice versa

Oncentra	Distance from original to re-exported (mm)		Distance from re-exported to original (mm)		Number of RTSS vertices	
Structure	Average	Maximum	Average	Maximum	Original	Re-exported
Object A	0	0	0	0	100	100
Object B	0	0	0	0	100	100
Object C	0	0	0	0	14420	13994
Object D	0	0	0	0	14420	14207
Object E	0	0	0	0	2400	2400
Object F	0	0	0	0	20000	19806
Esophagus	$5x10^{-4}$	0.08	$2x10^{-7}$	$7x10^{-7}$	13217	12783
Heart	$3x10^{-3}$	0.35	$2x10^{-7}$	$7x10^{-7}$	14415	12978
Lung_L	$9x10^{-4}$	0.17	$2x10^{-7}$	$7x10^{-7}$	35080	33451
Lung_R	$6x10^{-4}$	0.36	$2x10^{-7}$	$7x10^{-7}$	39065	38434
SpinalCord	$2x10^{-4}$	0.06	$2x10^{-7}$	$6x10^{-7}$	10744	10387

Pinnacle	Distance from original to re-exported (mm)		Distance from re-exported to original (mm)		Number of RTSS vertices	

Structure	Average	Maximum	Average	Maximum	Original	Re-exported
Object A	0.28	0.28	0.19	0.21	100	10400
Object B	0.28	0.28	0.19	0.21	100	10400
Object C	0.32	0.43	0.33	0.43	14420	14280
Object D	0.32	0.43	0.33	0.43	14420	14280
Object E	0.25	0.68	0.24	0.69	2400	21032
Object F	0.22	0.66	0.23	0.69	20000	20960
Esophagus	0.19	0.57	0.25	0.59	13217	3484
Heart	0.20	0.56	0.24	0.77	14415	9120
Lung_L	0.23	36.52	0.26	0.85	35080	23477
Lung_R	0.21	9.88	0.25	0.88	39065	27523
SpinalCord	0.19	0.52	0.26	0.59	10744	2861

ProKnow	Distance from original to re-exported (mm)		Distance from re-exported to original (mm)		Number of RTSS vertices	
Structure	Average	Maximum	Average	Maximum	Original	Re-exported
Object A	0	0	0	0	100	100
Object B	0	0	0	0	100	100
Object C	0	0	0	0	14420	280
Object D	0	0	0	0	14420	280
Object E	5×10^{-16}	0	0	0	2400	400
Object F	0	0	0	0	20000	400
Esophagus	4×10^{-4}	3×10^{-3}	2×10^{-4}	7×10^{-4}	13217	6389
Heart	3×10^{-4}	2×10^{-3}	2×10^{-4}	7×10^{-4}	14415	13250
Lung_L	3×10^{-4}	3×10^{-3}	2×10^{-4}	7×10^{-4}	35080	31769
Lung_R	3×10^{-4}	3×10^{-3}	2×10^{-4}	7×10^{-4}	39065	34850
SpinalCord	4×10^{-4}	3×10^{-3}	2×10^{-4}	7×10^{-4}	10744	5748

ProSoma	Distance from original to re-exported (mm)		Distance from re-exported to original (mm)		Number of RTSS vertices	
Structure	Average	Maximum	Average	Maximum	Original	Re-exported
Object A	0	0	0	0	100	100
Object B	0	0	0	0	100	100
Object C	2×10^{-5}	2×10^{-3}	0	0	14420	14420
Object D	2×10^{-5}	2×10^{-3}	0	0	14420	14420
Object E	9×10^{-16}	0	9×10^{-16}	0	2400	2400
Object F	0	0	0	0	20000	20000
Esophagus	2×10^{-3}	7×10^{-3}	2×10^{-3}	7×10^{-3}	13217	13217
Heart	2×10^{-3}	7×10^{-3}	2×10^{-3}	7×10^{-3}	14415	14415
Lung_L	2×10^{-3}	7×10^{-3}	2×10^{-3}	7×10^{-3}	35080	35080
Lung_R	2×10^{-3}	7×10^{-3}	2×10^{-3}	7×10^{-3}	39065	39065
SpinalCord	2×10^{-3}	7×10^{-3}	2×10^{-3}	7×10^{-3}	10744	10744

Raystation 2D	Distance from original to re-exported (mm)	Distance from re-exported to original (mm)	Number of RTSS vertices

Structure	Average	Maximum	Average	Maximum	Original	Re-exported
Object A	0	0	0	0	100	100
Object B	0	0	0	0	100	100
Object C	0	0	0	0	14420	14016
Object D	0	0	0	0	14420	14016
Object E	$4x10^{-7}$	$7x10^{-6}$	$4x10^{-7}$	$7x10^{-6}$	2400	2380
Object F	0	0	0	0	20000	19608
Esophagus	$2x10^{-4}$	0.09	$2x10^{-5}$	$6x10^{-5}$	13217	13019
Heart	$4x10^{-4}$	0.06	$2x10^{-5}$	$7x10^{-5}$	14415	14011
Lung_L	$2x10^{-4}$	0.09	$2x10^{-5}$	$7x10^{-5}$	35080	34643
Lung_R	$2x10^{-4}$	0.13	$2x10^{-5}$	$7x10^{-5}$	39065	38600
SpinalCord	$1x10^{-4}$	0.09	$2x10^{-5}$	$6x10^{-5}$	10744	10567

Raystation 3D	Distance from re-exported to original (mm)		Distance from original to re-exported (mm)		Number of RTSS vertices	
Structure	Average	Maximum	Average	Maximum	Original	Re-exported
Object A	0.40	0.50	0.02	0.18	100	284
Object B	0.40	0.50	0.02	0.18	100	284
Object C	0.01	0.45	0.03	0.20	14420	454
Object D	0.01	0.45	0.03	0.20	14420	454
Object E	0.10	0.75	0.08	0.48	2400	3366
Object F	0.03	0.75	0.08	0.48	20000	3366
Esophagus	0.05	0.37	0.03	0.31	13217	2012
Heart	0.04	0.41	0.05	0.51	14415	3851
Lung_L	0.09	36.37	0.05	0.63	35080	11069
Lung_R	0.06	12.41	0.05	0.74	39065	12181
SpinalCord	0.06	0.21	0.03	0.13	10744	2141

Velocity	Distance from re-exported to original (mm)		Distance from original to re-exported (mm)		Number of RTSS vertices	
Structure	Average	Maximum	Average	Maximum	Original	Re-exported
Object A	0.07	0.07	0.29	0.30	100	225
Object B	0.07	0.07	0.29	0.30	100	225
Object C	0.17	0.55	0.20	0.29	14420	315
Object D	0.17	0.55	0.20	0.29	14420	315
Object E	0.23	0.94	0.18	0.34	2400	996
Object F	0.19	0.94	0.18	0.34	20000	996
Esophagus	0.16	0.49	0.27	0.49	13217	1849
Heart	0.16	0.49	0.29	0.49	14415	4397
Lung_L	0.15	0.49	0.27	0.49	35080	12557
Lung_R	0.15	0.57	0.27	0.49	39065	14756
SpinalCord	0.16	0.49	0.25	0.49	10744	1598

S2 Distribution of differences from original contour

The plots in this section show the distribution of distances from the vertices on the original contour to the re-exported contour. Abrupt changes occur for the synthetic shape where the sampling of contour vertices was sparse. For the clinical shapes, the axis has been split to show the tail of the distribution. Note, that the range on the right-hand subplot varies with the organ

Object A

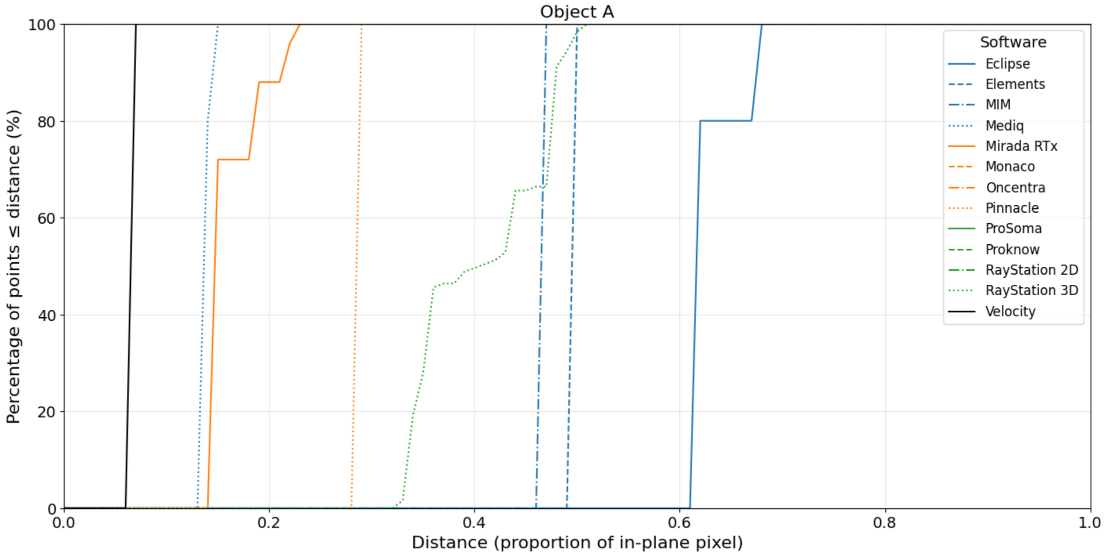

Object B

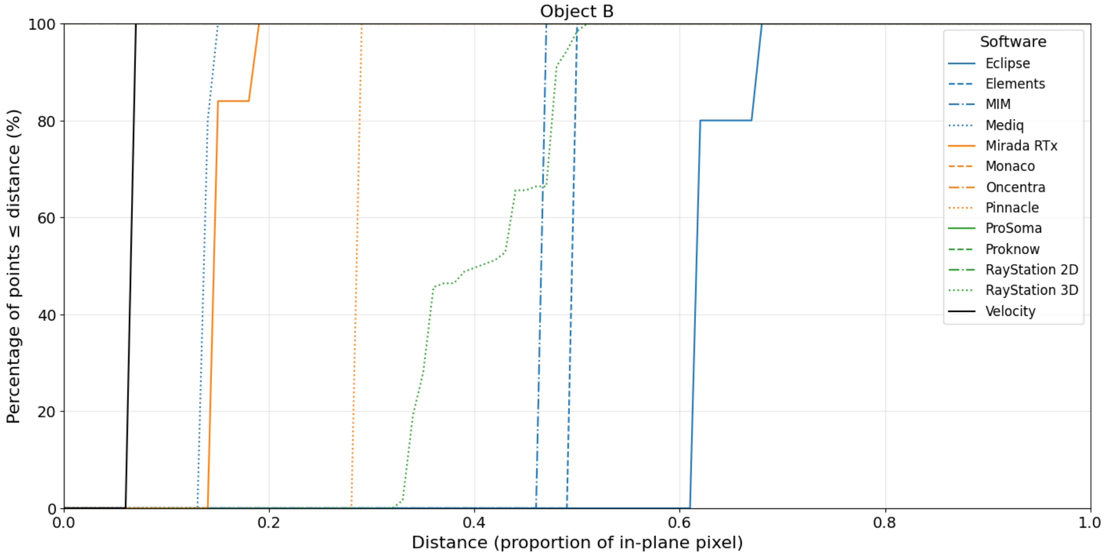

Object C

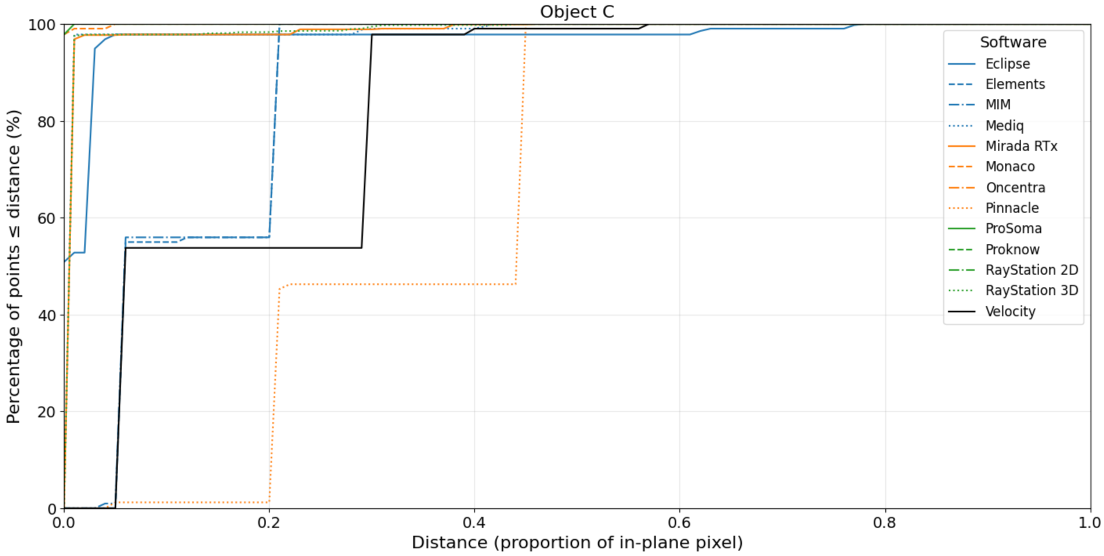

Object D

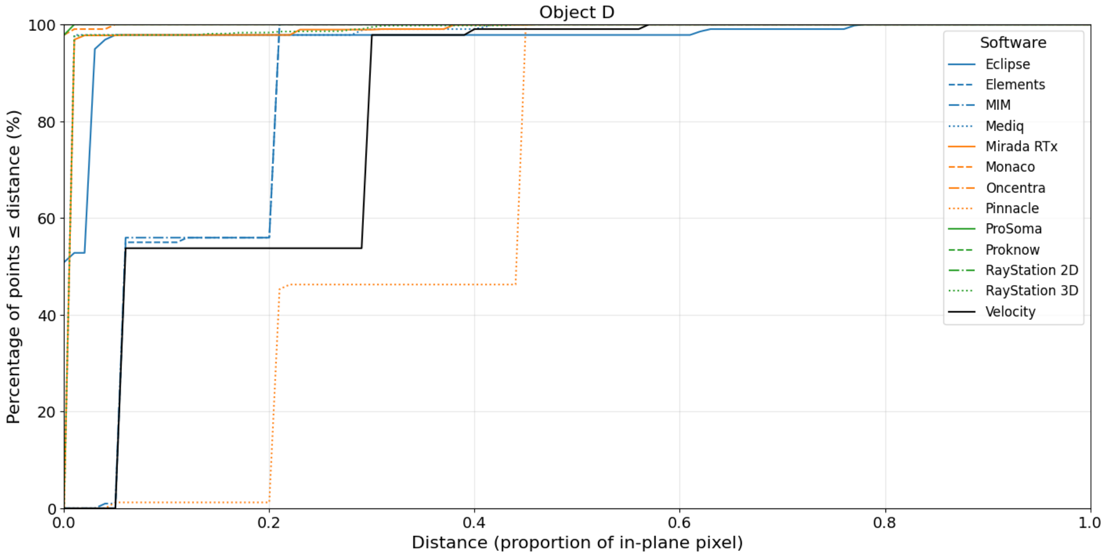

Object E

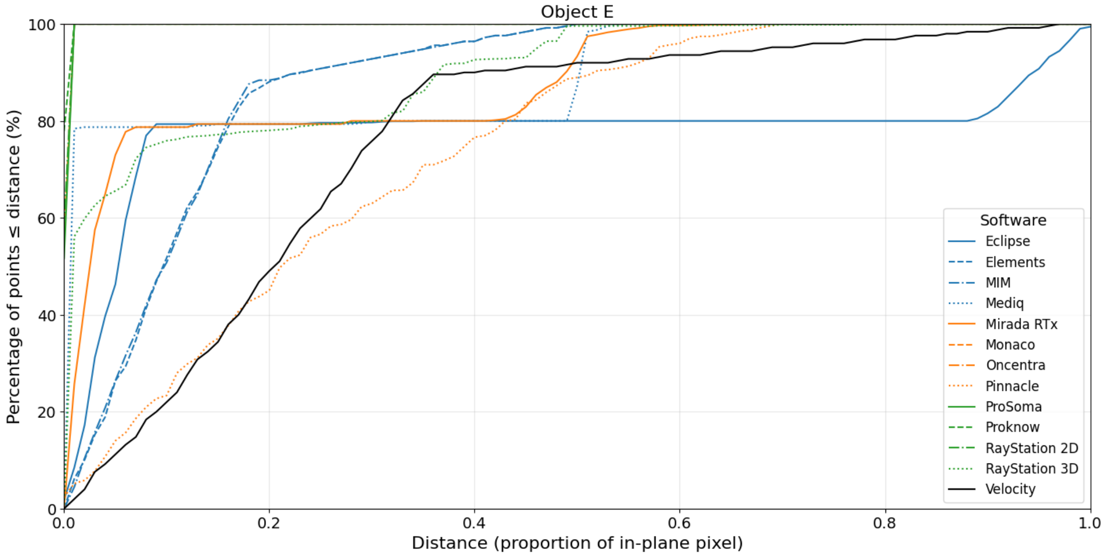

Object F

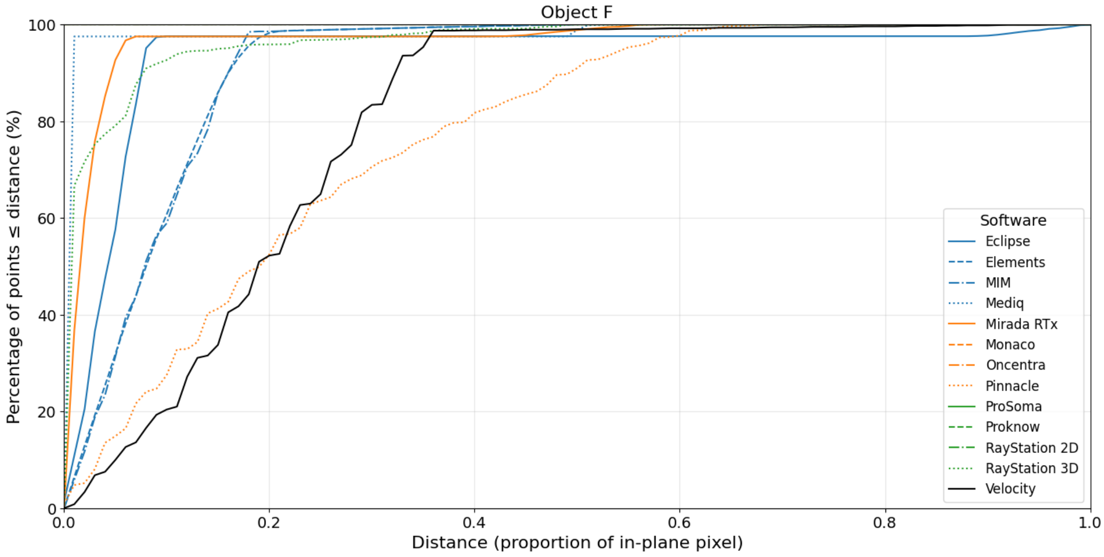

Esophagus

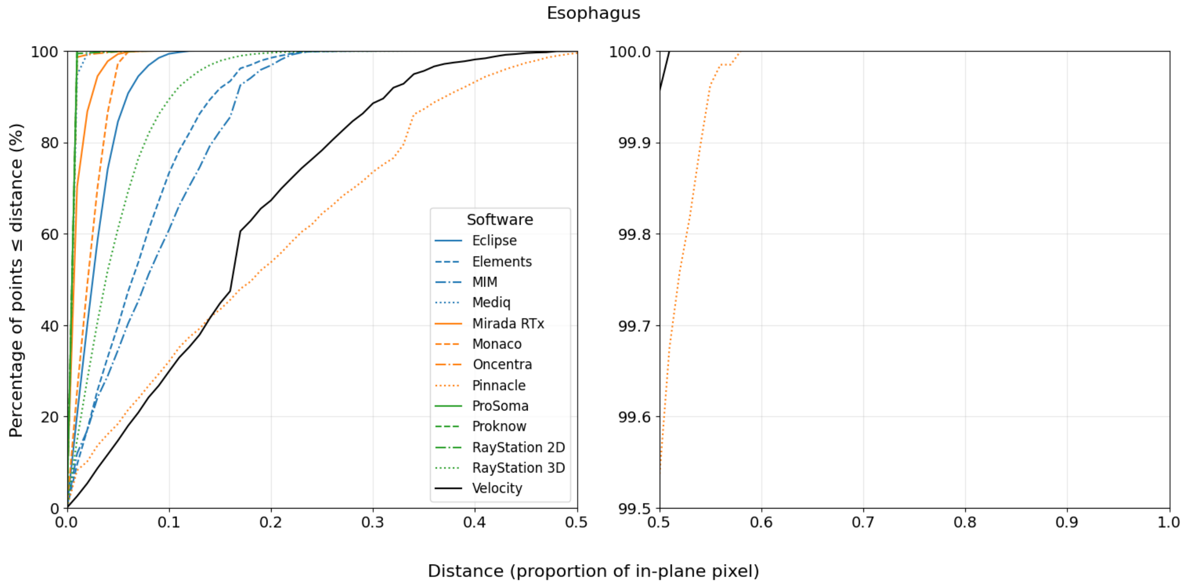

Heart

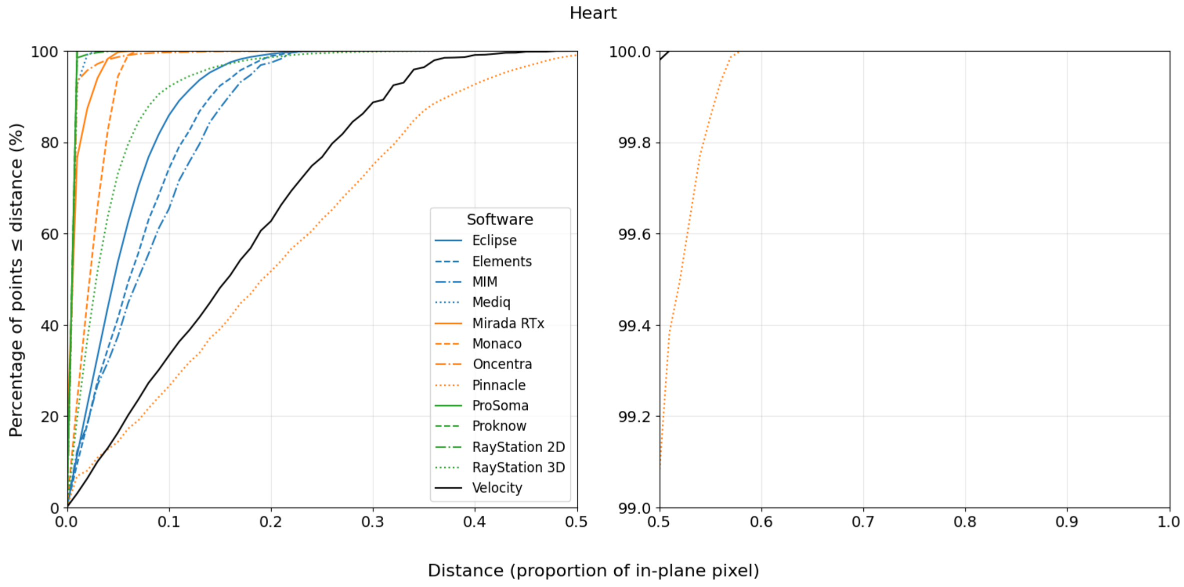

Lung_L

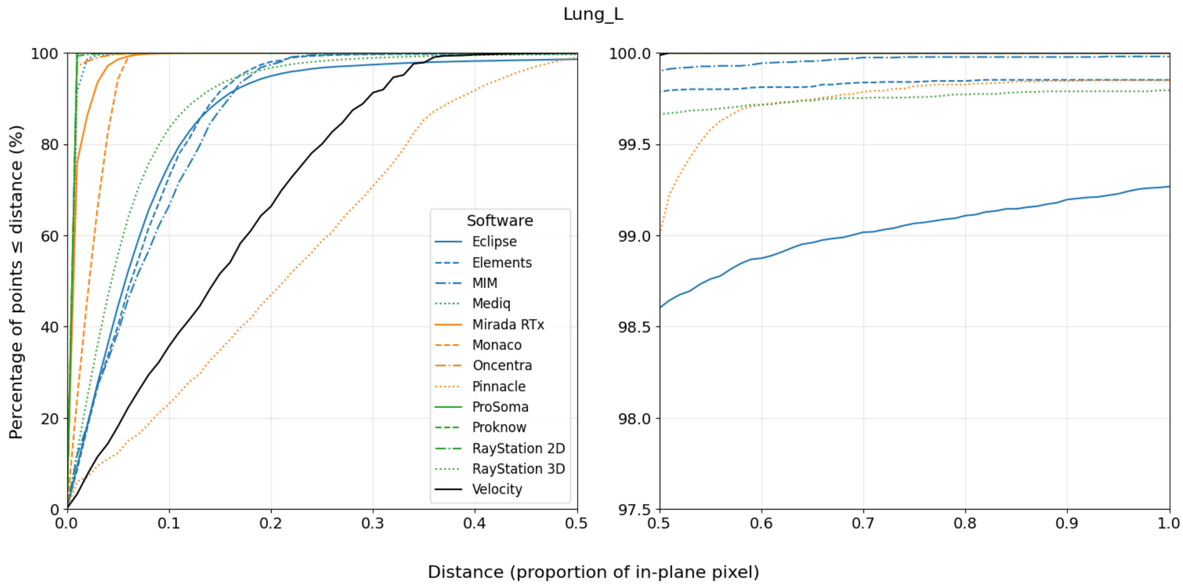
Lung_L
Percentage of points ≤ distance (%)
Distance (proportion of in-plane pixel)
Software
Eclipse
Elements
MIM
Mediq
Mirada RTx
Monaco
Oncentra
Pinnacle
ProSoma
Proknow
RayStation 2D
RayStation 3D
Velocity

Lung_R

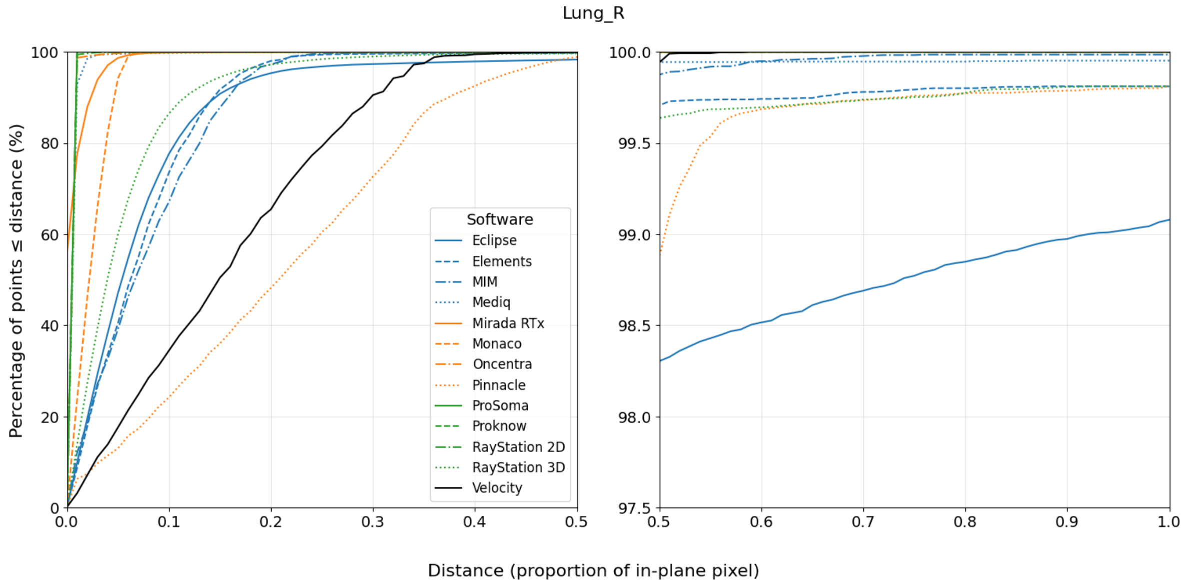
Lung_R
Percentage of points ≤ distance (%)
Distance (proportion of in-plane pixel)
Software
Eclipse
Elements
MIM
Mediq
Mirada RTx
Monaco
Oncentra
Pinnacle
ProSoma
Proknow
RayStation 2D
RayStation 3D
Velocity

SpinalCord

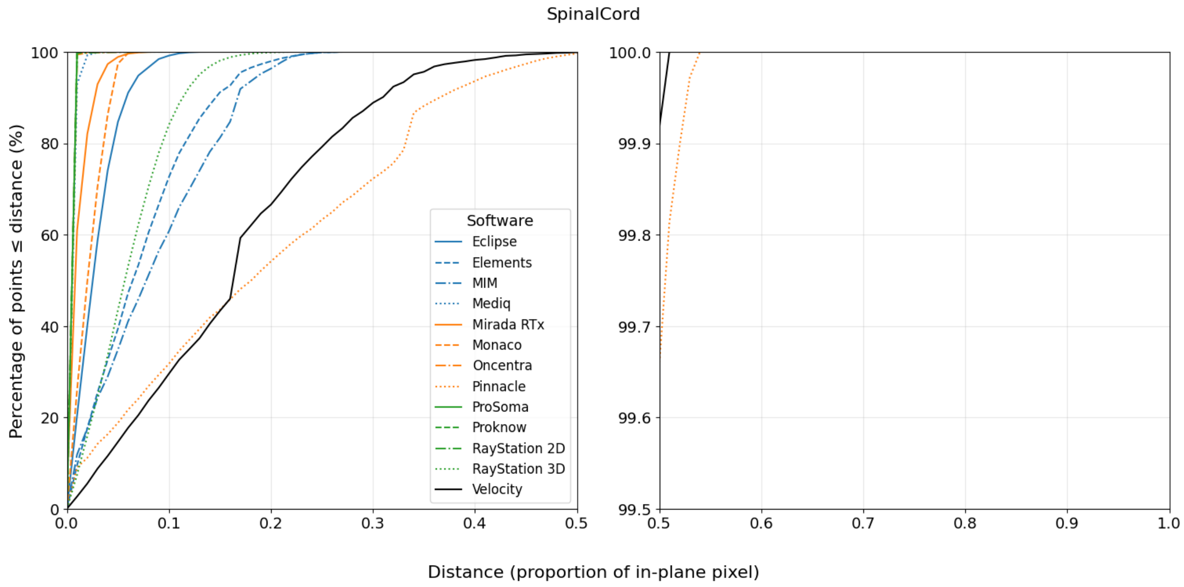
SpinalCord
Percentage of points ≤ distance (%)
Distance (proportion of in-plane pixel)
Software
Eclipse
Elements
MIM
Mediq
Mirada RTx
Monaco
Oncentra
Pinnacle
ProSoma
Proknow
RayStation 2D
RayStation 3D
Velocity

S3 Impact of system on contouring measures

The tables in this section give the values of the contouring similarity measures calculated between the re-exported contour and the original reference contour for each system. The reference value is calculated between the original test contour and the original reference contour. Thus, the only cause of the difference in calculation is the process of re-exporting the test contour from each system.

vDSC	Synthetic dataset structures						Challenge dataset structures				
	Object A	Object B	Object C	Object D	Object E	Object F	Esophagus	Heart	Lung_L	Lung_R	SpinalCord
Reference value	0.980	0.980	0.754	0.754	0.487	0.483	0.763	0.952	0.976	0.959	0.511
Eclipse	0.979	0.979	0.754	0.754	0.487	0.483	0.762	0.952	0.975	0.959	0.514
Elements	0.984	0.984	0.752	0.752	0.487	0.483	0.763	0.952	0.976	0.959	0.509
MIM	0.984	0.984	0.752	0.752	0.487	0.483	0.763	0.952	0.976	0.959	0.510
Mediq	0.980	0.980	0.754	0.754	0.487	0.483	0.763	0.952	0.976	0.959	0.510
Mirada RTx	0.980	0.980	0.754	0.754	0.487	0.483	0.763	0.952	0.976	0.959	0.510
Monaco	0.980	0.980	0.754	0.754	0.487	0.483	0.763	0.952	0.976	0.959	0.511
Oncentra	0.980	0.980	0.754	0.754	0.487	0.483	0.763	0.952	0.976	0.959	0.511
Pinnacle	0.984	0.984	0.756	0.756	0.488	0.484	0.756	0.952	0.974	0.958	0.524
ProSoma	0.980	0.980	0.754	0.754	0.487	0.483	0.763	0.952	0.976	0.959	0.511
Proknow	0.980	0.980	0.754	0.754	0.487	0.483	0.763	0.952	0.976	0.959	0.511
RayStation 2D	0.980	0.980	0.754	0.754	0.487	0.483	0.763	0.952	0.976	0.959	0.511
RayStation 3D	0.980	0.980	0.754	0.754	0.487	0.483	0.765	0.952	0.976	0.959	0.501
Velocity	0.975	0.975	0.756	0.756	0.488	0.483	0.760	0.952	0.975	0.958	0.514

2DHD	Synthetic dataset structures						Challenge dataset structures				
	Object A	Object B	Object C	Object D	Object E	Object F	Esophagus	Heart	Lung_L	Lung_R	SpinalCord
Reference value	1.58	1.64	5.00	5.00	51.76	51.76	7.72	31.25	93.62	113.03	8.98
Eclipse	1.60	1.60	5.02	5.02	51.72	51.72	7.82	31.07	73.42	110.71	8.93
Elements	1.29	1.29	5.20	5.20	51.86	51.93	7.68	31.13	74.26	110.62	8.85
MIM	1.29	1.31	5.20	5.20	51.90	51.90	7.73	31.15	93.05	112.63	8.79
Mediq	1.54	1.58	5.00	5.00	51.76	51.76	7.72	31.25	93.61	113.03	8.98
Mirada RTx	1.52	1.57	5.01	5.01	51.75	51.80	7.72	31.24	93.59	113.03	8.98
Monaco	1.58	1.64	5.00	5.00	51.76	51.76	7.68	31.23	93.60	113.03	8.94
Oncentra	1.58	1.64	5.00	5.00	51.76	51.76	7.72	31.25	93.62	113.03	8.98
Pinnacle	1.33	1.38	5.20	5.20	51.96	51.77	7.96	31.58	74.74	110.80	8.64
ProSoma	1.58	1.64	5.00	5.00	51.76	51.76	7.72	31.25	93.62	113.03	8.98
Proknow	1.58	1.64	5.00	5.00	51.76	51.76	7.72	31.25	93.62	113.03	8.98
RayStation 2D	1.58	1.64	5.00	5.00	51.76	51.76	7.72	31.25	93.62	113.03	8.98
RayStation 3D	1.50	1.50	5.00	5.00	51.83	51.83	7.64	31.36	74.15	110.58	9.01
Velocity	1.81	1.85	5.06	5.06	52.07	52.07	8.10	31.18	94.04	113.41	9.04

95% 2DHD	Synthetic dataset structures						Challenge dataset structures				
	Object A	Object B	Object C	Object D	Object E	Object F	Esophagus	Heart	Lung_L	Lung_R	SpinalCord
Reference value	1.50	1.50	5.00	5.00	49.80	50.38	4.70	9.00	3.09	12.82	5.26
Eclipse	1.60	1.60	5.02	5.02	49.78	50.36	4.72	8.97	2.80	12.94	5.22
Elements	1.29	1.29	5.20	5.20	49.80	50.39	4.66	8.95	2.96	12.78	5.22
MIM	1.29	1.29	5.20	5.20	49.80	50.39	4.66	8.89	2.99	12.73	5.17
Mediq	1.51	1.51	5.00	5.00	49.81	50.39	4.70	9.00	3.09	12.82	5.26
Mirada RTx	1.50	1.50	5.01	5.01	49.81	50.39	4.69	9.00	3.06	12.82	5.26
Monaco	1.50	1.50	5.00	5.00	49.80	50.38	4.70	8.99	3.10	12.81	5.25
Oncentra	1.50	1.50	5.00	5.00	49.80	50.38	4.70	9.00	3.09	12.82	5.26
Pinnacle	1.29	1.29	5.20	5.20	49.69	50.40	4.81	8.82	3.05	12.74	5.17
ProSoma	1.50	1.50	5.00	5.00	49.80	50.38	4.70	9.00	3.09	12.82	5.26
Proknow	1.50	1.50	5.00	5.00	49.80	50.38	4.70	9.00	3.09	12.82	5.26
RayStation 2D	1.50	1.50	5.00	5.00	49.80	50.38	4.70	9.00	3.09	12.82	5.26
RayStation 3D	1.50	1.50	5.00	5.00	49.82	50.40	4.63	8.94	2.89	12.83	5.32
Velocity	1.78	1.78	5.06	5.06	49.75	50.34	4.73	8.79	3.18	12.63	5.13

50% 2DHD	Synthetic dataset structures						Challenge dataset structures				
	Object A	Object B	Object C	Object D	Object E	Object F	Esophagus	Heart	Lung_L	Lung_R	SpinalCord
Reference value	0.81	0.90	5.00	5.00	38.30	38.86	1.01	1.39	0.44	0.53	2.85
Eclipse	0.69	0.73	5.00	5.00	38.50	39.07	1.02	1.42	0.47	0.55	2.83
Elements	0.53	0.62	5.06	5.06	38.36	38.92	1.01	1.38	0.44	0.52	2.84
MIM	0.56	0.64	5.06	5.06	38.34	38.90	1.02	1.37	0.44	0.51	2.82
Mediq	0.79	0.88	5.00	5.00	38.40	38.97	1.01	1.39	0.45	0.53	2.85
Mirada RTx	0.78	0.88	4.99	4.99	38.39	38.95	1.00	1.39	0.44	0.53	2.86
Monaco	0.81	0.90	5.00	5.00	38.30	38.86	1.00	1.39	0.44	0.53	2.85
Oncentra	0.81	0.90	5.00	5.00	38.30	38.86	1.01	1.39	0.44	0.53	2.85
Pinnacle	0.60	0.68	4.57	4.57	38.18	38.60	1.07	1.40	0.48	0.54	2.71
ProSoma	0.81	0.90	5.00	5.00	38.30	38.86	1.01	1.39	0.44	0.53	2.85
Proknow	0.81	0.90	5.00	5.00	38.30	38.86	1.01	1.39	0.44	0.53	2.85
RayStation 2D	0.81	0.90	5.00	5.00	38.30	38.86	1.01	1.39	0.44	0.53	2.85
RayStation 3D	0.73	0.82	5.00	5.00	38.42	38.99	0.99	1.38	0.44	0.53	2.90
Velocity	1.10	1.20	4.89	4.84	38.34	38.88	1.04	1.39	0.46	0.53	2.80

2DMSD	Synthetic dataset structures						Challenge dataset structures				
	Object A	Object B	Object C	Object D	Object E	Object F	Esophagus	Heart	Lung_L	Lung_R	SpinalCord
Reference value	1.00	1.00	4.75	4.75	36.11	36.64	1.38	2.42	0.97	1.79	2.95
Eclipse	1.06	1.06	4.76	4.76	36.25	36.79	1.39	2.44	0.94	1.76	2.93
Elements	0.80	0.80	4.86	4.86	36.14	36.67	1.37	2.41	0.95	1.77	2.95
MIM	0.80	0.80	4.86	4.86	36.13	36.67	1.36	2.39	0.95	1.77	2.93
Mediq	1.00	1.00	4.75	4.75	36.18	36.72	1.38	2.42	0.97	1.79	2.96
Mirada RTx	1.00	1.00	4.75	4.75	36.17	36.71	1.38	2.42	0.96	1.79	2.96
Monaco	1.00	1.00	4.75	4.75	36.11	36.64	1.38	2.42	0.97	1.79	2.95
Oncentra	1.00	1.00	4.75	4.75	36.11	36.64	1.38	2.42	0.97	1.79	2.95
Pinnacle	0.80	0.80	4.63	4.63	36.04	36.50	1.40	2.41	0.99	1.80	2.83
ProSoma	1.00	1.00	4.75	4.75	36.11	36.64	1.38	2.42	0.97	1.79	2.95
Proknow	1.00	1.00	4.75	4.75	36.11	36.64	1.38	2.42	0.97	1.79	2.95
RayStation 2D	1.00	1.00	4.75	4.75	36.11	36.64	1.38	2.42	0.97	1.79	2.95
RayStation 3D	1.00	1.00	4.75	4.75	36.20	36.74	1.36	2.41	0.94	1.76	3.00
Velocity	1.29	1.29	4.65	4.65	36.13	36.67	1.37	2.40	0.99	1.81	2.89

3DMSD	Synthetic dataset structures						Challenge dataset structures				
	Object A	Object B	Object C	Object D	Object E	Object F	Esophagus	Heart	Lung_L	Lung_R	SpinalCord
Reference value	0.56	0.55	6.77	6.76	29.21	29.65	1.45	1.59	0.60	1.25	2.84
Eclipse	0.56	0.54	6.78	6.77	29.27	29.67	1.45	1.61	0.61	1.26	2.82
Elements	0.43	0.41	6.84	6.83	29.24	29.64	1.43	1.59	0.60	1.25	2.85
MIM	0.37	0.35	6.90	6.89	29.24	29.64	1.43	1.59	0.60	1.25	2.85
Mediq	0.53	0.51	6.77	6.76	29.26	29.66	1.44	1.59	0.60	1.25	2.84
Mirada RTx	0.53	0.51	6.77	6.76	29.25	29.66	1.44	1.59	0.60	1.25	2.85
Monaco	0.56	0.55	6.77	6.76	29.21	29.65	1.45	1.59	0.60	1.25	2.84
Oncentra	0.56	0.55	6.77	6.76	29.21	29.65	1.45	1.59	0.60	1.25	2.84
Pinnacle	0.43	0.41	6.75	6.74	29.09	29.49	1.47	1.60	0.63	1.26	2.72
ProSoma	0.56	0.55	6.77	6.76	29.21	29.65	1.45	1.59	0.60	1.25	2.84
Proknow	0.56	0.55	6.77	6.76	29.21	29.61	1.45	1.59	0.60	1.25	2.84
RayStation 2D	0.56	0.55	6.77	6.76	29.21	29.64	1.45	1.59	0.60	1.25	2.84
RayStation 3D	0.55	0.53	6.77	6.76	29.30	29.71	1.42	1.59	0.59	1.24	2.90
Velocity	0.67	0.65	6.71	6.70	29.21	29.61	1.44	1.59	0.61	1.26	2.80

nAPL @100%	Synthetic dataset structures						Challenge dataset structures				
	Object A	Object B	Object C	Object D	Object E	Object F	Esophagus	Heart	Lung_L	Lung_R	SpinalCord
Reference value	0.495	0.496	0.980	0.980	1.000	1.000	0.476	0.643	0.147	0.181	0.994
Eclipse	0.495	0.495	0.980	0.980	1.000	1.000	0.478	0.651	0.159	0.194	0.992
Elements	0.493	0.494	0.980	0.980	1.000	1.000	0.470	0.637	0.145	0.181	0.993
MIM	0.493	0.494	0.980	0.980	1.000	1.000	0.462	0.629	0.138	0.175	0.993
Mediq	0.495	0.496	0.980	0.980	1.000	1.000	0.476	0.643	0.148	0.181	0.994
Mirada RTx	0.495	0.496	0.980	0.980	1.000	1.000	0.476	0.643	0.147	0.181	0.994
Monaco	0.495	0.496	0.980	0.980	1.000	1.000	0.472	0.643	0.146	0.181	0.994
Oncentra	0.495	0.496	0.980	0.980	1.000	1.000	0.476	0.643	0.147	0.181	0.994
Pinnacle	0.493	0.494	0.980	0.980	1.000	1.000	0.480	0.636	0.166	0.205	0.986
ProSoma	0.495	0.496	0.980	0.980	1.000	1.000	0.476	0.643	0.147	0.181	0.994
Proknow	0.495	0.496	0.980	0.980	1.000	1.000	0.476	0.643	0.147	0.181	0.994
RayStation 2D	0.495	0.496	0.980	0.980	1.000	1.000	0.476	0.643	0.147	0.181	0.994
RayStation 3D	0.495	0.496	0.980	0.980	1.000	1.000	0.471	0.640	0.146	0.178	0.995
Velocity	0.498	0.499	0.980	0.980	1.000	1.000	0.468	0.630	0.154	0.193	0.988

sDSC @100%	Synthetic dataset structures						Challenge dataset structures				
	Object A	Object B	Object C	Object D	Object E	Object F	Esophagus	Heart	Lung_L	Lung_R	SpinalCord
Reference value	0.751	0.751	0.027	0.027	0.000	0.000	0.510	0.462	0.878	0.831	0.011
Eclipse	0.750	0.750	0.027	0.027	0.000	0.000	0.508	0.455	0.874	0.827	0.012
Elements	0.755	0.757	0.027	0.027	0.000	0.000	0.514	0.462	0.878	0.831	0.011
MIM	0.759	0.878	0.027	0.027	0.000	0.000	0.515	0.464	0.877	0.831	0.011
Mediq	0.752	0.753	0.027	0.027	0.000	0.000	0.510	0.462	0.877	0.831	0.011
Mirada RTx	0.752	0.753	0.027	0.027	0.000	0.000	0.511	0.462	0.878	0.831	0.011
Monaco	0.751	0.751	0.027	0.027	0.000	0.000	0.510	0.463	0.878	0.831	0.011
Oncentra	0.750	0.750	0.027	0.027	0.000	0.000	0.510	0.463	0.878	0.831	0.011
Pinnacle	0.756	0.756	0.027	0.027	0.000	0.000	0.488	0.457	0.861	0.814	0.018
ProSoma	0.751	0.751	0.027	0.027	0.000	0.000	0.511	0.462	0.878	0.830	0.011
Proknow	0.749	0.751	0.027	0.027	0.000	0.000	0.510	0.462	0.878	0.831	0.011
RayStation 2D	0.751	0.751	0.027	0.027	0.000	0.000	0.510	0.462	0.878	0.831	0.011
RayStation 3D	0.752	0.754	0.027	0.027	0.000	0.000	0.516	0.466	0.881	0.833	0.010
Velocity	0.746	0.746	0.027	0.027	0.000	0.000	0.508	0.461	0.871	0.825	0.015

S4 Difference in tolerance-based measures at different tolerances

The plots in this section show the impact of re-exporting the test contour on the nAPL and sDSC measures at different tolerances (given as a percentage of in-plane pixel spacing). When comparing to the original test contour, the expected value of nAPL is 0 and sDSC is 1. For figures showing the measures between the re-exported test contour and the original reference contour, the absolute values of the measures and their difference from the value calculated using the original test contour are shown.

nAPL between original test contour and re-exported test contour

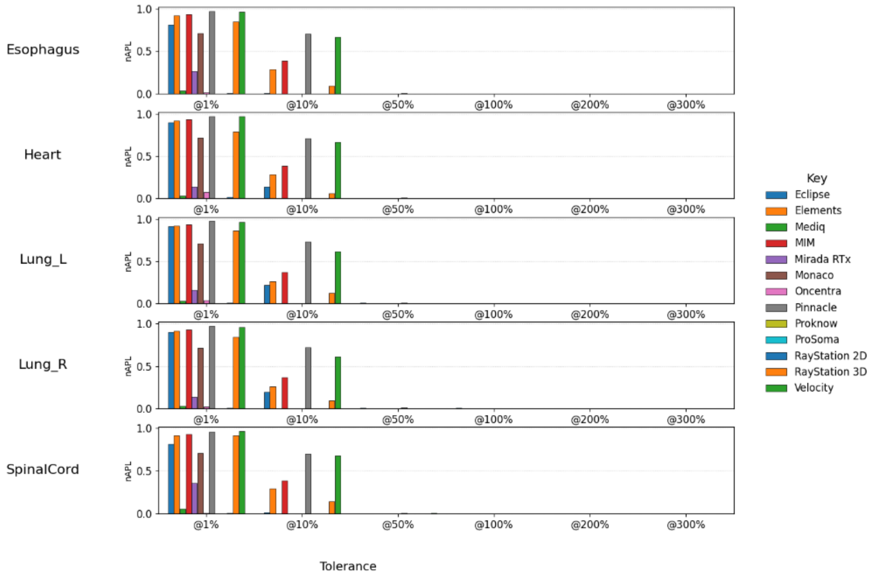

nAPL between original reference contour and re-exported test contour

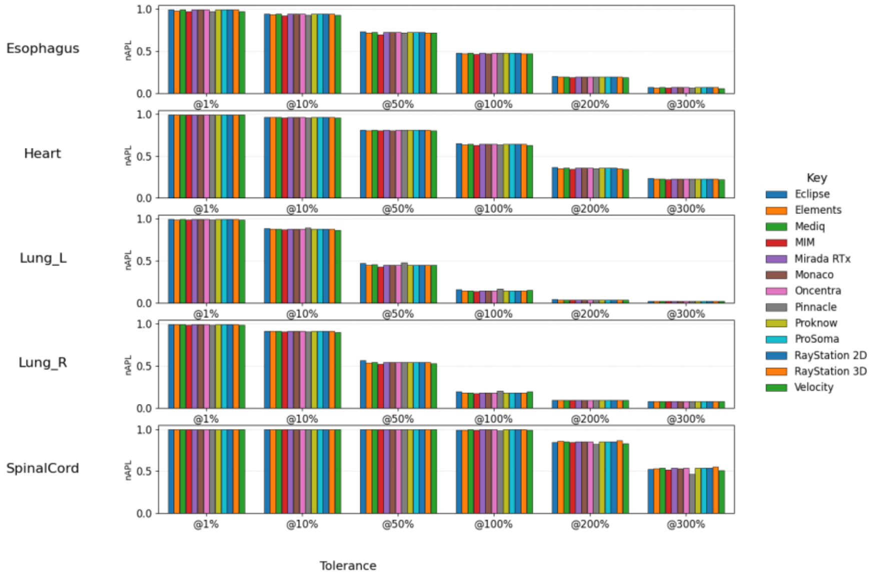

Difference in nAPL: value with re-exported test contour – value with original rest contour

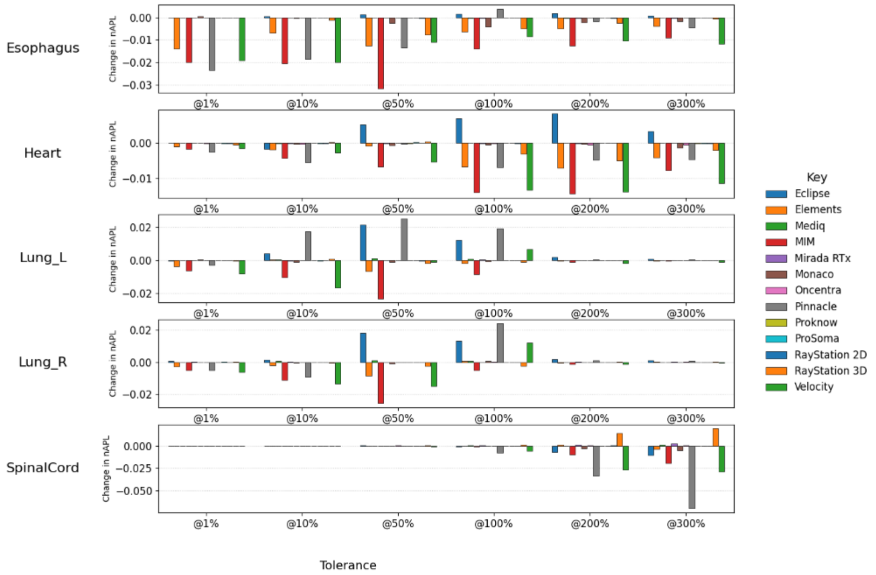

sDSC between original test contour and re-exported test contour

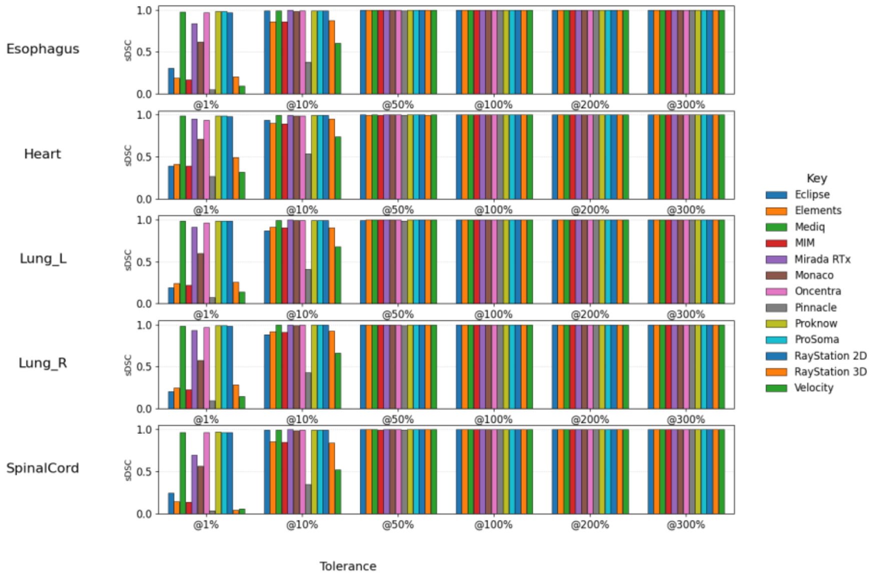

sDSC between original reference contour and re-exported test contour

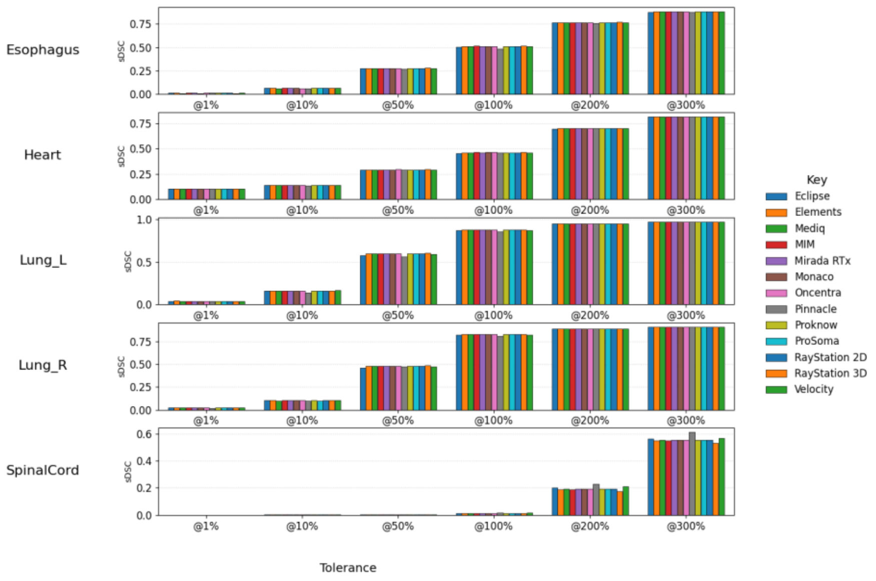

Difference in sDSC: value with re-exported test contour – value with original rest contour

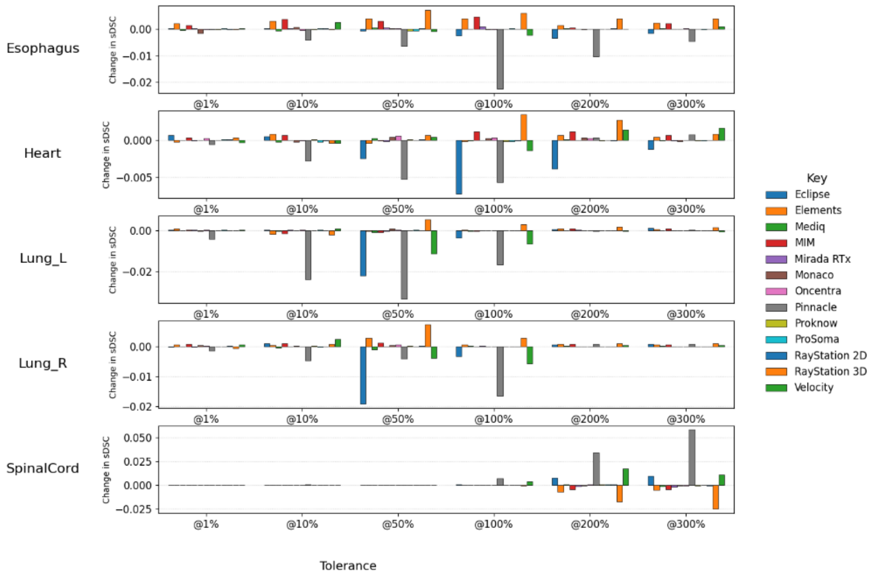

S5 Precision of contour position recording

In DICOM RTSS, the contour positions are recorded as a list of decimal string representation. Some of the small differences in contour boundary position can be explained by the precision to which the various systems output these point locations. The table below gives the maximum number of decimal places used for any point coordinate by each system for the contours of the Lung_R

Software	Maximum precision (Number of decimal places)
**INPUT (WorkflowBox, Mirada)**	22
**Eclipse**	2
**Elements**	6
**Mediq**	6
**MIM**	3
**MiradaRTx**	22
**Monaco**	1
**Oncentra**	6
**Pinnacle**	2
**ProKnow**	3
**ProSoma**	2
**RayStation2D**	20
**RayStation3D**	9
**Velocity**	4

S6 Impact of image resolution

The main study did not investigate the impact of image resolution on contour displacement but scaled the displacement relative to the in-plane pixel spacing to put the displacement in context and allow for systems that may use a CT-resolution internal representation for the structures. A small post-hoc analysis was performed to determine if this scaling was appropriate.

The CT image was sub-sampled to by 1.5 times and 2.0 times leading to images of 388 and 256 pixels in-plane size respectively. Out-of-plane resampling was not performed, such that the initial contours (stored in real-world coordinates in DICOM) would still be applicable to the same image slices.

The data was processed with the same loading, non-editing action, saving procedure as in the main study using Eclipse. Eclipse was chosen as a system for which a moderate amount of contour displacement in the main study, and visual inspection of the contours seemed to suggest a resolution-based resampling.

The figures below show the cumulative histogram of displacement for the various CT resolutions, with the displacement shown in mm and relative to the voxel size.

This limited investigation shows that for some organs the displacement is resolution depended, but also that the behaviour can vary between organs. For the Esophagus and SpinalCord, normalising by the in-plane pixel motion results in the approximately the same curve. Whereas for the displacement cumulative histogram for subsampling by a factor of two is similar to the original resolution cumulative histogram for the Lungs and Heart, while the subsampling by a factor of 1.5 is still yields a resolution depended displacement for these organs.

Esophagus

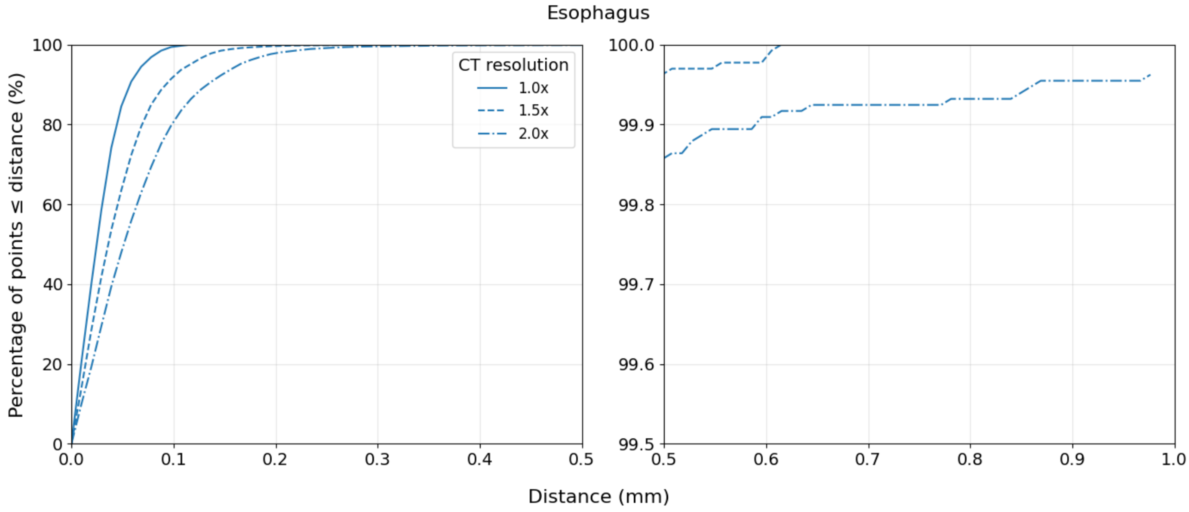
Esophagus
Percentage of points ≤ distance (%)
100
80
60
40
20
0
0.0
0.1
0.2
0.3
0.4
0.5
CT resolution
1.0x
1.5x
2.0x
100.0
99.9
99.8
99.7
99.6
99.5
0.5
0.6
0.7
0.8
0.9
1.0
Distance (mm)

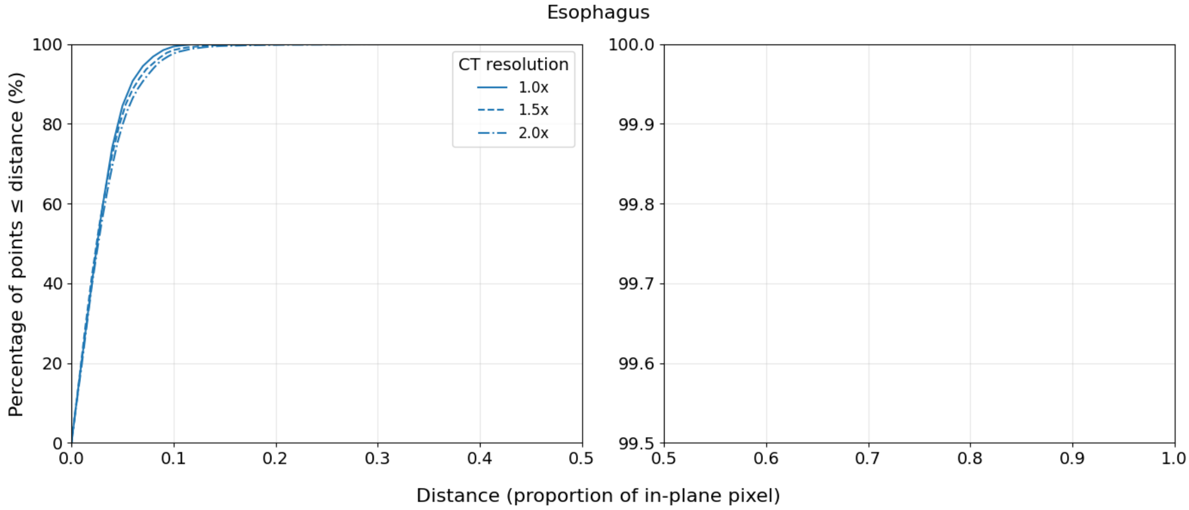
Esophagus
Percentage of points ≤ distance (%)
100
80
60
40
20
0
0.0
0.1
0.2
0.3
0.4
0.5
CT resolution
1.0x
1.5x
2.0x
100.0
99.9
99.8
99.7
99.6
99.5
0.5
0.6
0.7
0.8
0.9
1.0
Distance (proportion of in-plane pixel)

Heart

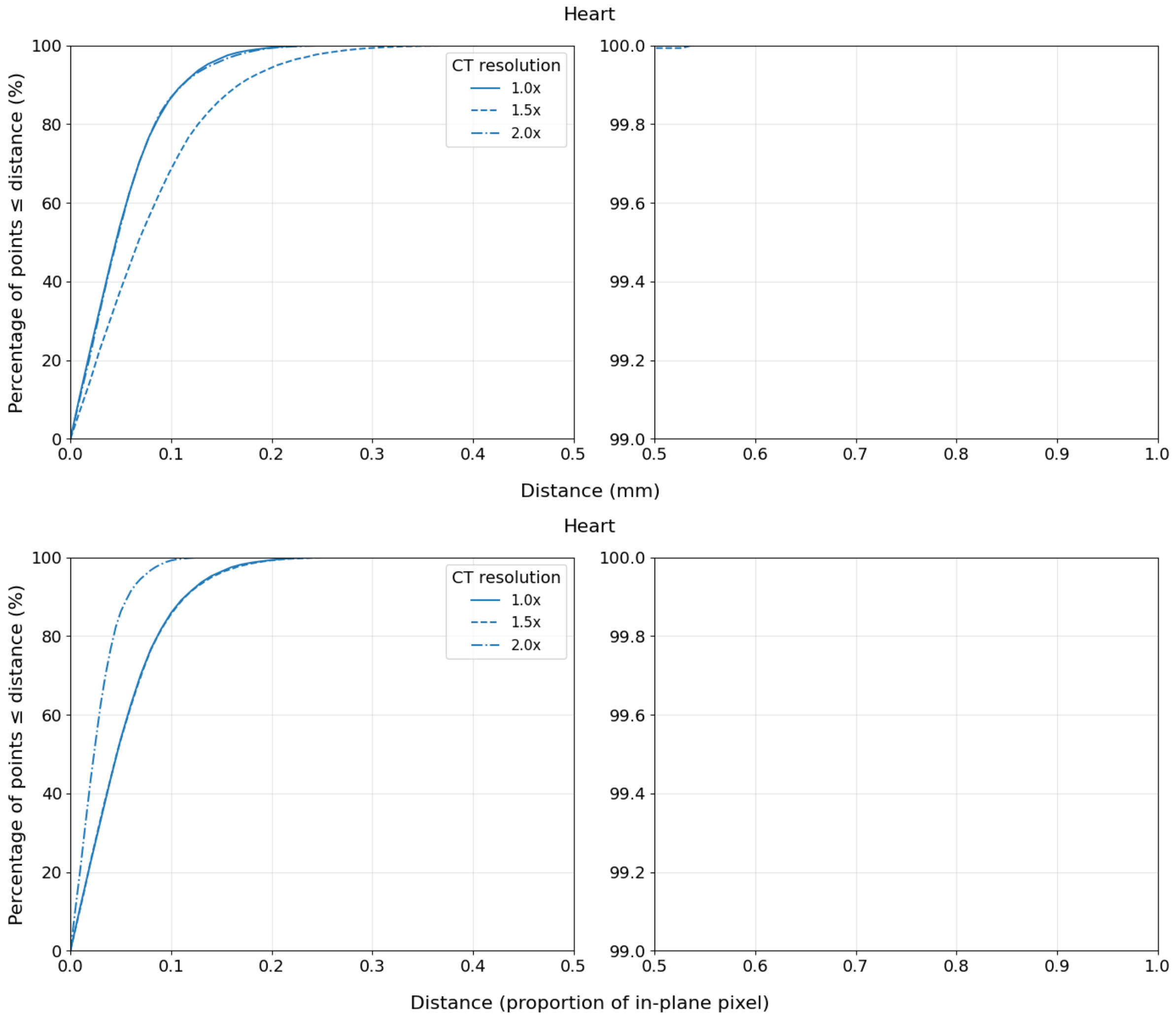
Heart
CT resolution
1.0x
1.5x
2.0x
Percentage of points ≤ distance (%)
100
80
60
40
20
0
0.0
0.1
0.2
0.3
0.4
0.5
100.0
99.8
99.6
99.4
99.2
99.0
0.6
0.7
0.8
0.9
1.0
Distance (mm)
Heart
Distance (proportion of in-plane pixel)

Lung_L

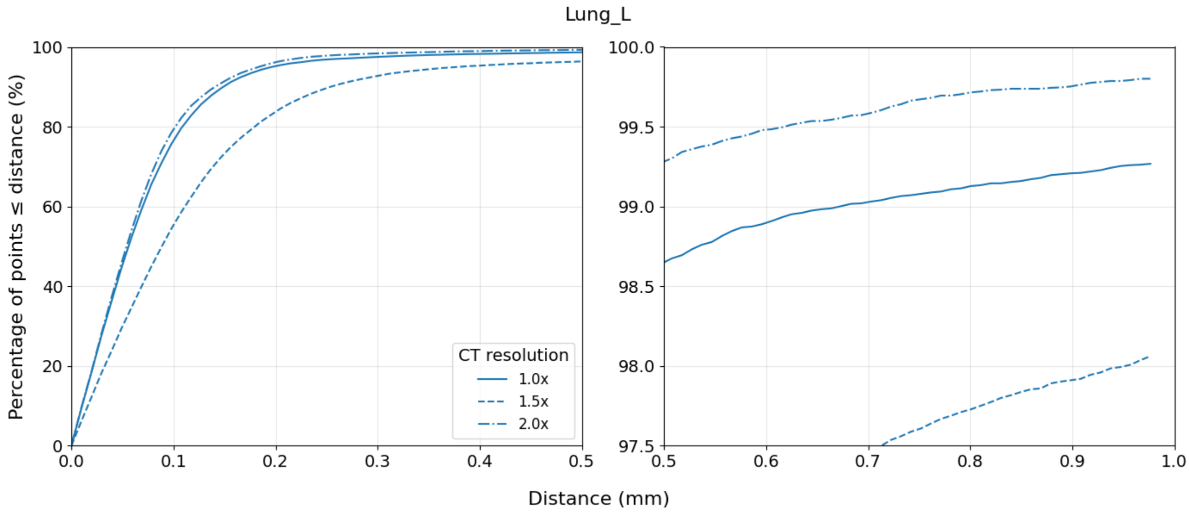
Lung_L
Percentage of points ≤ distance (%)
Distance (mm)
CT resolution
1.0x
1.5x
2.0x

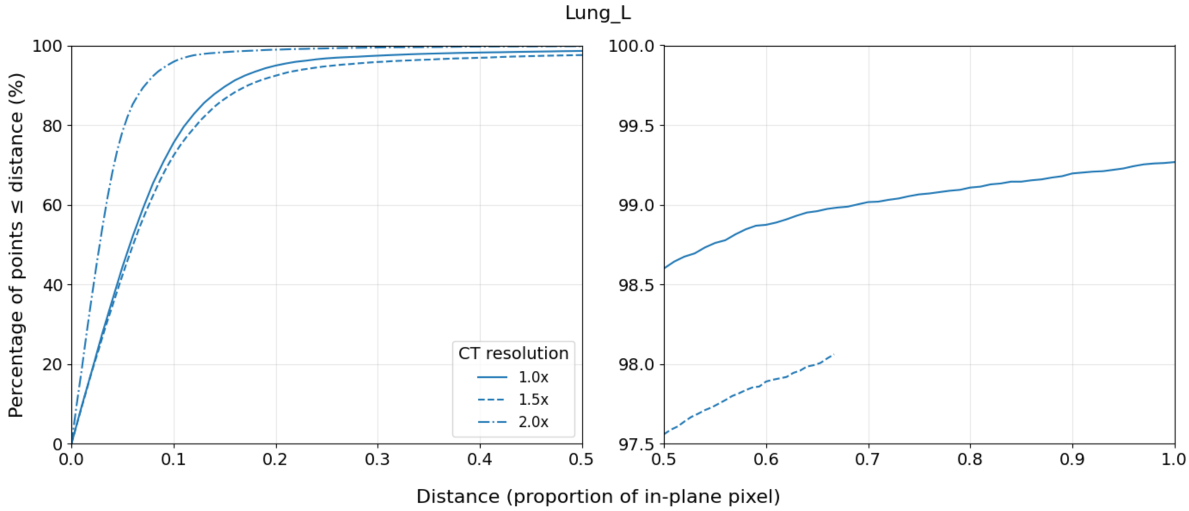
Lung_L
Percentage of points ≤ distance (%)
Distance (proportion of in-plane pixel)
CT resolution
1.0x
1.5x
2.0x

Lung_R

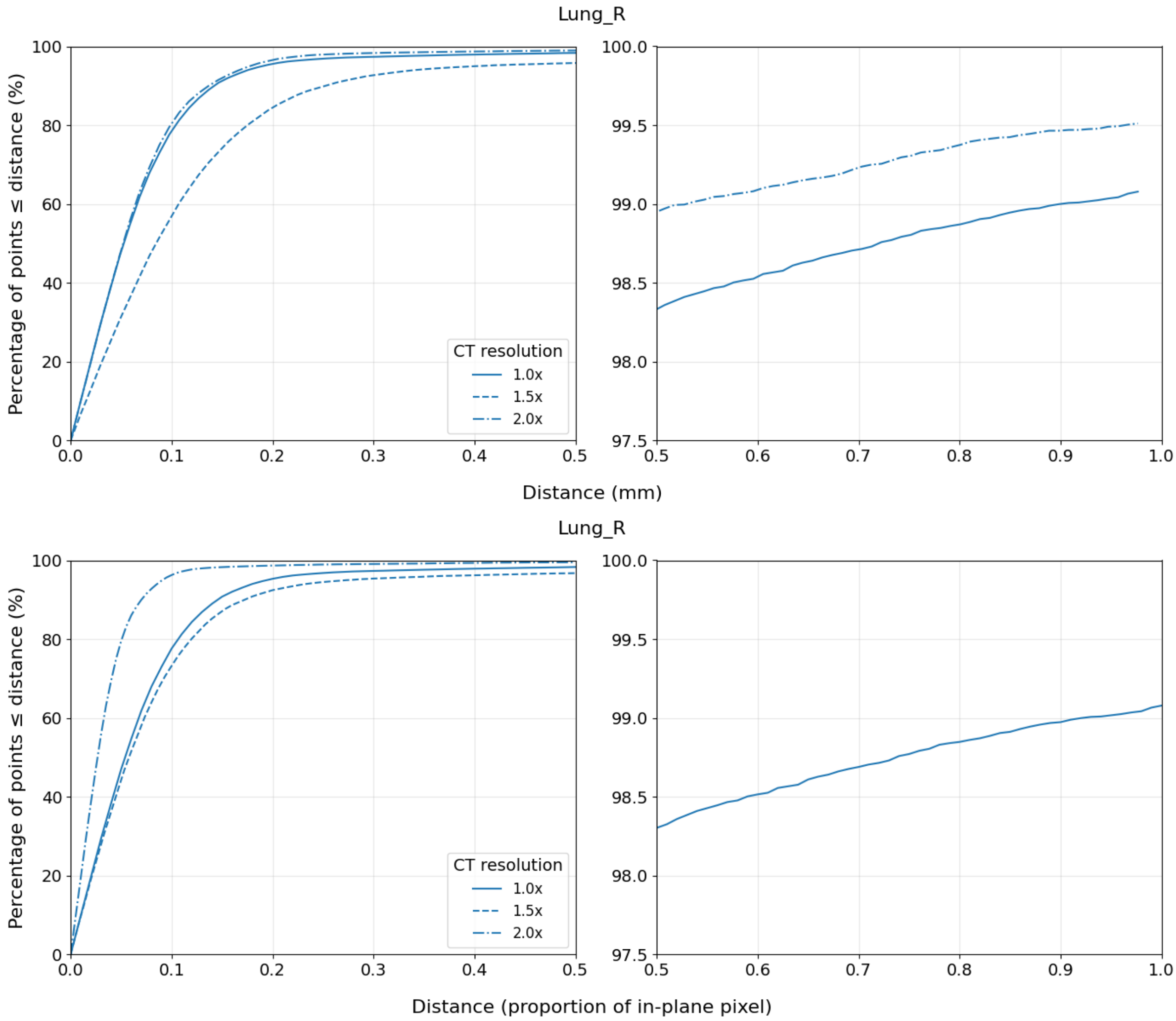
Lung_R
Percentage of points ≤ distance (%)
CT resolution
1.0x
1.5x
2.0x
Distance (mm)
Lung_R
Percentage of points ≤ distance (%)
CT resolution
1.0x
1.5x
2.0x
Distance (proportion of in-plane pixel)

SpinalCord

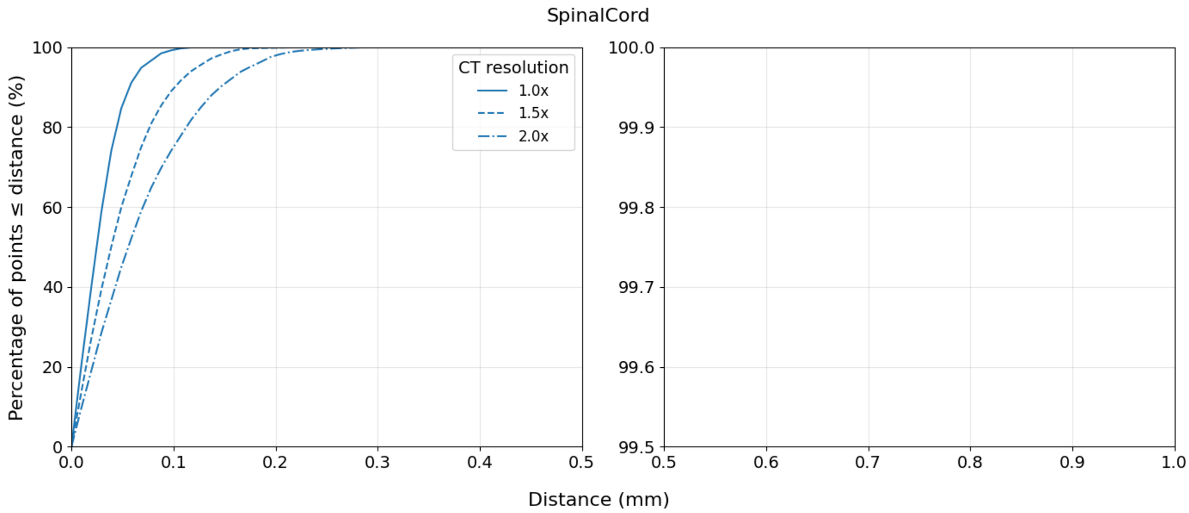
SpinalCord
Percentage of points ≤ distance (%)
CT resolution
1.0x
1.5x
2.0x
Distance (mm)

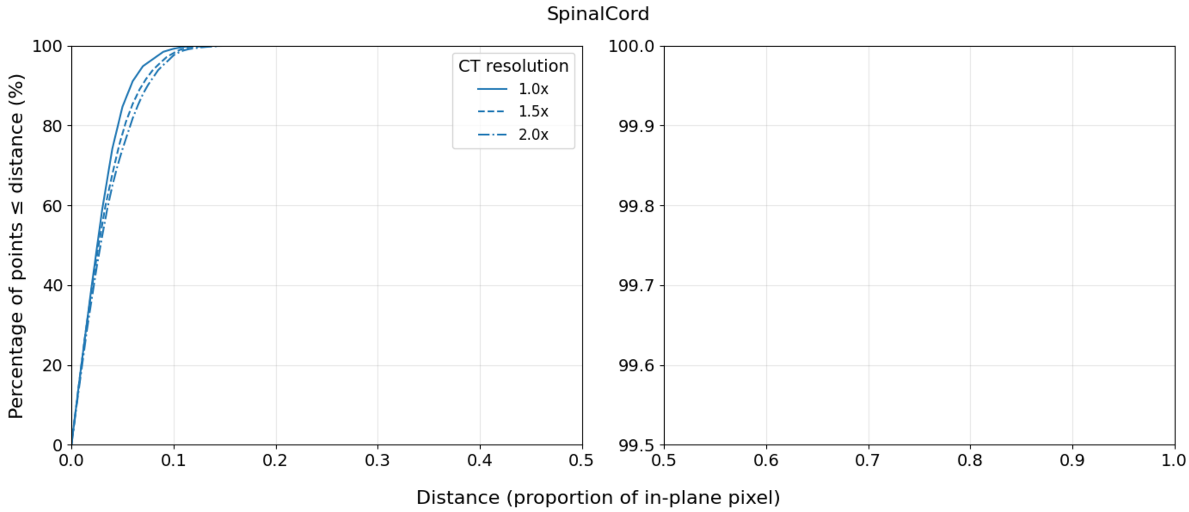
SpinalCord
Percentage of points ≤ distance (%)
CT resolution
1.0x
1.5x
2.0x
Distance (proportion of in-plane pixel)
